# Switch chemistry at cryogenic conditions: quantum tunnelling under electric fields†

**DOI:** 10.1039/d0sc06295b

**Published:** 2020-12-15

**Authors:** Omer Kirshenboim, Alexander Frenklah, Sebastian Kozuch

**Affiliations:** Department of Chemistry, Ben-Gurion University of the Negev Beer-Sheva 841051 Israel kozuch@bgu.ac.il

## Abstract

While the influence of intramolecular electric fields is a known feature in enzymes, the use of oriented external electric fields (EEF) to enhance or inhibit molecular reactivity is a promising topic still in its infancy. Herein we will explore computationally the effects that EEF can provoke in simple molecules close to the absolute zero, where quantum tunnelling (QT) is the sole mechanistic option. We studied three exemplary systems, each one with different reactivity features and known QT kinetics: π bond-shifting in pentalene, Cope rearrangement in semibullvalene, and cycloreversion of diazabicyclohexadiene. The kinetics of these cases depend both on the field strength and its direction, usually giving subtle but remarkable changes. However, for the cycloreversion, which suffers large changes on the dipole through the reaction, we also observed striking results. Between the effects caused by the EEF on the QT we observed an inversion of the Arrhenius equation, deactivation of the molecular fluxionality, and stabilization or instantaneous decomposition of the system. All these effects may well be achieved, literally, at the flick of a switch.

## Abstract 2

Adding an external electric field to reactions driven by quantum mechanical tunneling brings a whole new dimension to the idea of switch chemistry.

## Graphical abstract

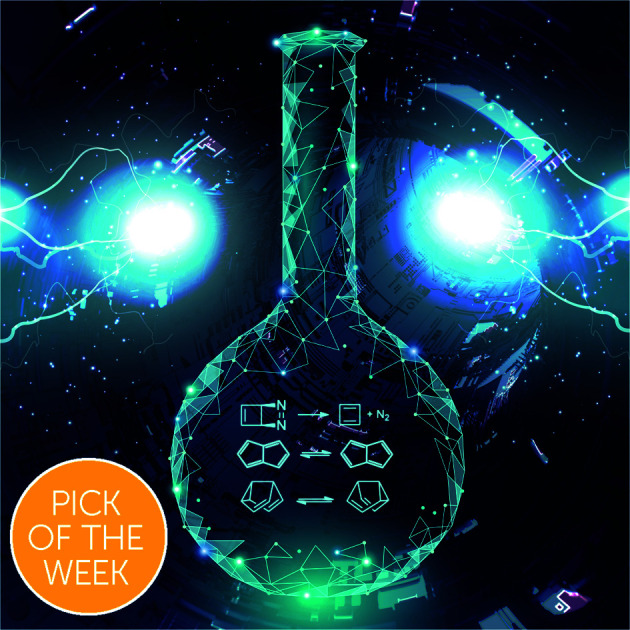

## Introduction

To tune and control chemical reactions there are two main approaches: the chemical and the physical. The former involves playing with substituents, solvents, pH, protecting groups, *etc.* The latter is based on external influences, where temperature is by far the most exploited one, photochemistry and electrochemistry coming in a second place, and many other fascinating external stimuli exist in more specific disciplines (pressure, mechanochemistry, sonochemistry, radiochemistry, magnetochemistry, *etc.*).^1^

In recent years a new type of physical interaction started to appear in the shape of External Electric Fields (EEF)^2–8^ (subjectively elected as one of “chemistry's next big thing” in C & EN^9^). The intrinsic molecular electrostatic effects have been known for some time, especially in enzymes.^10–12^ However, the effect of an external field and its chemical consequences was first developed as shy theoretical predictions in heme models^13–15^ and, due to the augmented charge separation at the transition state, in Diels–Alder reactions.^16^ Subsequently, a ground-breaking STM experimental work based on the previous theoretical work on Diels–Alder reactivity opened the doors for a more realistic chemistry of EEF outside the *in silico* bubble.^17^ From there, the EEF concept has seen many more (mostly theoretical) applications.^18–35^

According to IUPAC “A substance that increases the rate of a reaction without modifying the overall standard Gibbs energy change in the reaction; the process is called catalysis. The catalyst is both a reactant and product”.^36^ Therefore, EEF can be taken as a physical catalyst,^8,9,17,37–42^ but only in a weak sense of the term: opposed to a chemical catalyst the Δ*G*_r_ is affected by the field, so the overall standard Gibbs energy is not modified (one of IUPAC requirements for catalysts) only once the field is turned off. But more important, the field is not a reactant or a product, and not even a substance, also essential factors by IUPAC's definition. What is certain is that it can substantially increase (or inhibit) the rate of a reaction, and therefore we can safely call it a pseudo-catalyst. In that sense, we will try to understand and exploit the pseudo-catalytic EEF effect at extremely low temperatures.

The second topic that concerns us here is heavy-atom quantum tunnelling (QT). While hydrogen QT is widely known,^43^ heavy atom QT (*i.e.* for second row atoms^44^) is a relatively new phenomenon, as can be attested by the number of recent reviews and perspectives on the field.^45–50^ In simple terms, the tunnelling rate depends on the mass of the tunnelling particle (or the reduced mass, especially on reactions where the whole molecule is moving), on the barrier height, and, the most critical factor, the barrier width.^43,51,52^ Therefore, even atoms heavier than H can and will tunnel when the trajectory through the reaction is short, and the barrier height is not excessive. This effect is clearly seen at very low temperatures, where classical thermal reactions are impossible but ground-state temperature independent tunnelling can be accessible.

**Fig. 1:**
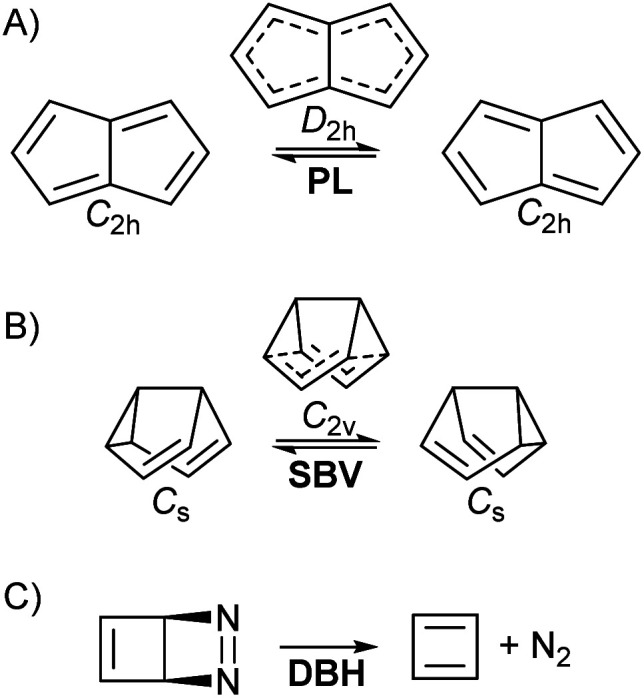
The three studied reactions (note that the symmetries may change when applying an EEF). (A) Pentalene (PL) π bond-shifting. (B) Semibullvalene (SBV) Cope rearrangement. (C) Diazabicyclohexadiene (DBH) degradation to cyclobutadiene and N_2_ in a “pseudo-*retro*-Diels–Alder” reaction.

Herein, as considered elsewhere,^53–56^ we will bring the two effects together: we will discuss and make theoretical predictions about the EEF impact on the QT rates of prototypical molecules in gas phase and cryogenic conditions. The selected molecules are three systems with experimentally and/or theoretically proven reactions by tunnelling:

• Pentalene (PL) π-bond shifting automerization,^57^ involving an isothermic symmetrical reaction coordinate with a degenerate double-well potential (Fig. 1A).

• Semibullvalene (SBV) Cope rearrangement,^53,58–61^ another degenerate reaction (Fig. 1B).

• Diazabicyclohexadiene (DBH) cycloreversion, similar to a retro-Diels–Alder reaction (Fig. 1C), suffering decomposition even at 0 K by quantum tunnelling instability.^50,62–64^

Knowing the current difficulties that building an experimental setup for such a project entails, this is more a proof of concept article than a protocol for applied chemistry. However, as we shall see the effects can be drastic, and therefore worth pursuing.

### Theoretical method

As explained elsewhere,^50,65^ there is a striking difference between the rates of reaction of gas phase systems (that is, individual molecules without any interaction with other molecules) and in cryogenic matrices, where the frozen “solvent” (typically a frozen noble gas^49^) can interact and affect the reactivity.^58,66^ Here we decided to simulate non-interacting gas phase reactions, unadulterated by any external effect (except for the EEF, obviously). While such sterile conditions is a sensible choice in a theoretical and academical sense, we must point out that experimental devices that generate ultracold non-interacting molecules are not uncommon, such as in the supersonic expansion methods.^67^

High accuracy in QT rates can only be achieved by employing highly accurate computational tunnelling methods on highly accurate potential energy surfaces (PES). Therefore, QT was computed with the small-curvature tunnelling method (SCT),^68–70^ a multidimensional technique built on top of the semi-classical canonical variational theory (CVT),^68,70,71^ including in our case quantization of the vibrational levels (QRST^72^) below 100 K. This method provides reliable results, but at a high computational cost. Unless specified, all the tunnelling included rate constants are at cryogenic conditions close to the absolute zero, *i.e.* ground state, temperature independent processes (below 20 K all the reactions already converged to this regime).

The selection of the underlying electronic method to compute energies and its derivatives was not an easy task. Usually a benchmark study permits the selection of a DFT functional with a relatively small basis set that matches the higher-level energy results. However, in this project the selected molecules were different enough to hinder the selection of an overall accurate functional. Therefore, we applied a double-level correction (the interpolated single point energy method^73^), where the DFT obtained PES was adjusted with three energy points (reactant, transition state and product) computed at the CCSD(T) level with a complete basis set extrapolation (obtained from cc-pVTZ and cc-pVQZ with *β* coefficients of 5 and 3 for the HF and post-HF correlation, respectively^74,75^). The basis set for the DFT must be small enough to permit the SCT computations in a reasonable time, but large enough to provide the electronic density flexibility that the strong polarization requires when adding an EEF. We found that 6-311G(d) reached a good balance (for the polarizability tests, which require large basis sets, we used Def2-QZVP). As said before, we found that no functional can work for all the reactions, and therefore any good functional with proven qualities for main group barriers would work. We choose M06-2x,^76^ which acted relatively well in our preliminary tests and in previous carbon QT projects.^62,77,78^ All the barrier heights (aka threshold energies) and reaction energies (Δ*E*^‡^ and Δ*E*_r_) presented here are CCSD(T)/CBS electronic energies plus zero-point energies at the M06-2x/6-311G(d) level.

All the quantum electronic computations were carried out with Gaussian,^79^ all the CVT/SCT rate constants with Polyrate,^80^ and Gaussrate^81^ was used for communication between them. For reproducibility purposes, due to the many Polyrate options, we included some input files in the ESI.†

An advantage of computational chemistry is that compared to experimental chemistry alterations and effects acting at the molecular level are extremely easy to implement. Adding an electric field is only a matter of a keyword (in Gaussian being, for instance, “field = *X* + 150”). This adds a potential term to the Hamiltonian corresponding to the field strength and direction, which will polarize the electron cloud. A value of 150 in the Gaussian input applies an electric field of 0.015 au, equivalent to 7.71 V nm^−1^. As in other works this value will be our maximum field strength, since it is extremely intense but technically still achievable^4^ (the other values used here were 2.57 and 5.14 V nm^−1^). While adding an electric field may seem straightforward (especially in highly symmetric structures), there is no standard in setting the direction of the field, and different conventions can give different results. For PL and SBV we used fields directed to the three natural axes that cross the highest symmetry structure (*i.e.* at the transition states). For DBH we aligned the field to the dipole moment of the transition state, to maximize the effect. In the figures below we show the EEF directions over each molecule; all the *XYZ* geometries can be found in the ESI.† Note that there is much confusion on the direction of the dipole moment and of the field;^4,27^ herein we use the chemical convention for the former (the dipole moment goes from positive to negative), and therefore a positive field as defined by Gaussian stabilizes the molecule when set parallel to the dipole.

Noteworthy, while in real gas phase the molecules will reorient setting the dipole moment parallel to the field,^54^ in STM^17^ and other experiments^42^ the reactant must be immobilized, opening the possibilities to multidirectional electric fields. For different reactions we will employ different approaches (multidirectional or unidirectional EEF), depending on the objective of the test. Also of note, neither Gaussian nor Polyrate automatically rotate and align the molecule as a function of the field, and therefore they act as if the molecule was effectively immobilized.

## Results and discussion

### Pentalene (PL)

The study of the π bond-shifting of PL^57^ was originally inspired by the grandparent of all heavy atom QT reactions: the automerization of cyclobutadiene.^45–47,82,83^ It was clear that the reaction can proceed by an extremely fast tunnelling even close to the absolute zero, with a computed rate constant of 1.1 × 10^8^ s^−1^ (see Table 1). Since then, many other similar reactions were tested, most of them showing the same signs of QT.^77,78,84–86^

**Table 1:** Field direction and strength (V nm^−1^), energies of activation (kJ mol^−1^), and QT rate constants from the ground state (*i.e.* approaching the absolute zero, in s^−1^) of PL and SBV^a^

EEF	PL		SBV	
	Δ*E*^‡^	*k*	Δ*E*^‡^	*k*
0.0	41.3	1.1 × 10^8^	29.1	8.2 × 10^−6^
*X* 2.6	41.0	1.1 × 10^8^	28.5	3.6 × 10^−5^
*X* 5.1	40.5	1.1 × 10^8^	27.0	1.4 × 10^−4^
*X* 7.7	39.2	1.3 × 10^8^	24.4	1.5 × 10^−3^
*Y* 2.6	40.5	1.1 × 10^8^	27.9	1.1 × 10^−4^
*Y* 5.1	38.1	1.6 × 10^8^	26.6	1.2 × 10^−3^
*Y* 7.7	34.0	2.9 × 10^8^	24.8	2.1 × 10^−3^
*Z* 2.6	40.9	2.5 × 10^7^	29.1	1.8 × 10^−5^
*Z* 5.1	40.1	3.6 × 10^5^	29.1	1.4 × 10^−5^
*Z* 7.7	38.9	1.0 × 10^3^	29.0	4.8 × 10^−6^

a In SBV with a field in the *Y* direction, the Δ*E*_r_ are −2.0, −4.0 and −6.3 kJ mol^−1^.

**Fig. 2:**
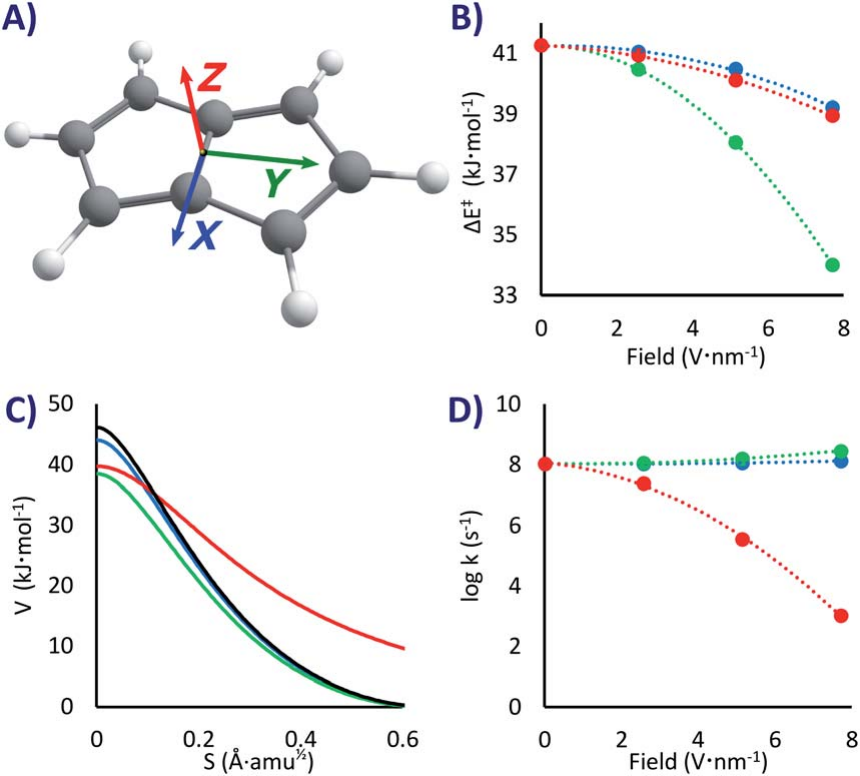
(A) PL and the colour coded directing axes at which the EEF was applied. (B) Δ*E*^‡^ as a function of the field strength. (C) Potential energies at the M06-2x/6-311G(d) level *vs.* the reaction mass-scaled coordinates (only one side of the symmetrical PES is shown); in black the profile without EEF, blue, green and red are with 7.7 V nm^−1^ fields in the *X*, *Y*, *Z* directions, respectively. (D) Tunnelling rate constants from the ground state *vs.* the field strength.

How much would EEF affect this PL degenerate rearrangement? To answer that question, we applied the field in three orthogonal directions, as shown in Fig. 2A. We did not expect a major rate change since the molecule is completely apolar, with a null dipole moment. However, the conjugation of the double bonds is significantly different in the minimum state compared to the fully conjugated transition state, a factor that might lower the threshold energy due to the enhanced electron mobility at the top of the barrier. In the same line, we expected some correlation between Δ*E*^‡^ and the polarizability difference between reactants and transition states (Δ*α* = *α*^TS^ − *α*^R^). Systems with larger polarizabilities have larger stabilization when a field is applied, as the dipole moment is enhanced.^87^ However, this is correct for static geometries, small fields and molecules with relatively defined dipole moment. For PL, the stabilization of the transition state and of the reactant as a function of the polarizabilities had significantly different slopes depending on the field direction (and even inversion of the slope, see Fig. S1 in the ESI†). As a consequence, while there was correlation with the field strength in any specific direction, there was no correlation when comparing between different field directions (there may be a more rational trend when analysing the non-isotropic polarizabilities, but that is beyond the scope of this work).

In addition, while we did not anticipate large geometrical changes with the field, in our experience small changes might bring unexpected outcomes. In pure thermal reactions the value of the Δ*E*^‡^ would provide enough data to test the change in the rate constant by applying transition state theory. In QT the barrier height is less critical (for TST the rate grows with the exponential of the energy, while for tunnelling it depends on the square root of the exponential^43,45,51,52^). But now the barrier width is a fundamental factor, such that the reaction might “cut corners” and not even pass through the transition state geometry. Moreover, in most cases a lower barrier also brings a narrower barrier.^50^

Noteworthy, as the electronic density is distorted by the field, the molecular symmetries vary; if in the absence of field the reaction goes from a *C*_2h_ reactant to a *D*_2h_ transition state, with the EEF in the *X* and *Y* directions it will go from *C*_s_ to *C*_2v_, and if applied in the *Z* direction it goes from *C*_2_ to *C*_2v_. In the latter the molecular planarity is broken, creating a concave compound that is exacerbated in the transition state, an effect with important consequences, as we shall see. In addition, with extremely fast tunnelling there is a ceiling to the rate given by the vibrational frequency of the system, where no system can react faster than the speed of impact into the barrier (of the order of 10^13^ s^−1^).^52^ Considering all these effects, we decided not to make further qualitative predictions and let the data do the talking.

With the EEFs applied in the *X* and *Y* directions the ground state QT rate constant is barely changing. As shown in Fig. 2D and Table 1, without any field we obtain *k*_0_ = 1.1 × 10^8^ s^−1^ (almost the same value of 2.2 × 10^8^ s^−1^ obtained in previous estimations^57^ with M06-2x/6-31G*). When activating the field at its maximum we get a rate acceleration of only 21% in *X* and 164% in Y (*k*_*X*+7.7_ = 1.3 × 10^8^, *k*_*Y*+7.7_ 2.9 × 10^8^ s^−1^). These relatively minor changes can be easily explained by the lowering of the activation energies with the field (by 2.0 kJ mol^−1^ with a 7.7 V nm^−1^ EEF in *X*, 7.3 kJ mol^−1^ in *Y*, and 2.3 kJ mol^−1^ in *Z*, see Fig. 2B and Table 1).

However, when setting the field in the *Z* direction the reaction rate is strongly reduced up to five orders of magnitude (*k*_*Z*+7.7_ = 1.0 × 10^3^ s^−1^), even when the field lowers the Δ*E*^‡^! As can be seen in Fig. 2C, the EEF in this case produces a lower but significantly wider barrier, that is a larger trajectory for the atoms, which, as explained above, is the main hindrance for a swift QT. As it seems, the molecule bends itself with the electric field perpendicular to its plane, a distortion that grows when approaching the transition state (see *XYZ* geometries in the ESI†). This variable change in concavity has a price in terms of atomic displacement, producing the counterintuitive kinetic effect of a slower reaction with a lower barrier, a kind of inverted Arrhenius effect.

In principle, as we raise the temperature the system should approach the classical regime and return to a normal kinetic behaviour (faster rates with lower barriers), including a critical temperature where the rate would be field independent (akin to the tunnelling control^88–91^). However, the tunnelling correction is large even at room temperature (*κ* ∼ 3000 without field, *κ* = 44 with a 7.7 V nm^−1^ EEF), and therefore the inverted Arrhenius effect appears, according to our model, beyond any reasonable testing temperature. Indeed, while at room temperature the Δ*G*^‡^ at the maximum 7.7 V nm^−1^ EEF in the *Z* direction is 3.2 kJ mol^−1^ lower than without field, the predicted rate is still 16 times slower. While an experimental test of this outcome would be challenging, if an EEF triggered inverted Arrhenius effect is observed, it will be an elegant verification of tunnelling in PL.

### Semibullvalene (SBV)

This system has a similar double-well potential as PL, but it has the advantage of having been thoroughly studied not only theoretically but also experimentally in cryogenic matrices.^53,58–61^ Previous SCT computations at the B3LYP/6-31G(d) level^60^ gave *k* = 1.4 × 10^−3^ s^−1^ below 40 K (Δ*H*^‡^ = 19 kJ mol^−1^), while the experiments in cryogenic matrices produced rate constants of the order of 10^−4^ s^−1^,^58,59^ depending on the matrix. This attests to the fortuitous error cancellation with B3LYP that provides accurate QT results only when compared to interacting environments (which tend to accelerate the reaction). Our CCSD(T)/M06-2x values, more akin to the value that would be obtained with non-interacting experiments, gives Δ*H*^‡^ = 29 kJ mol^−1^, and *k*_0_ = 8.2 × 10^−6^ s^−1^.

**Fig. 3:**
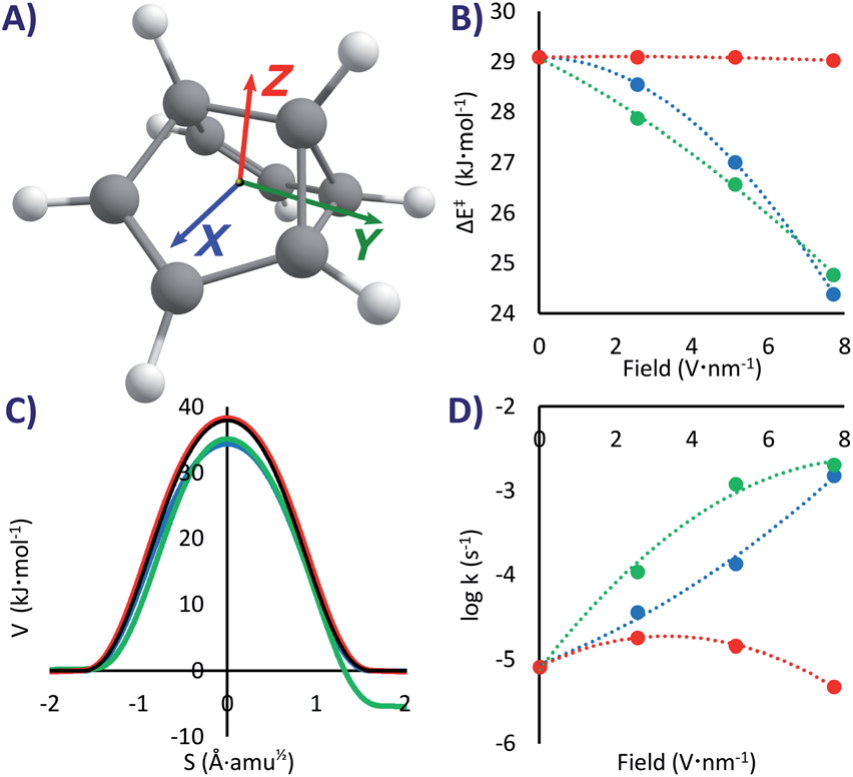
(A) SBV and the colour coded directing axes at which the EEF was applied. (B) Δ*E*^‡^ as a function of the field strength. (C) Potential energies at the M06-2x/6-311G(d) level *vs.* the reaction mass-scaled coordinates; in black the profile without EEF, blue, green and red are with 7.7 V nm^−1^ fields in the *X*, *Y*, *Z* directions, respectively; note the asymmetry with the EEF applied in the *Y* direction. (D) Tunnelling rate constants from the ground state *vs.* the field strength.

As with PL, due to the lack of a strong dipole moment, applying an EEF on SBV can have a significant but not substantial effect on the activation energy (our maximum change was of 4.7 kJ mol^−1^ in the *X* direction, a lowering of 16% compared to the no-field computation, see Table 1 and Fig. 3B). A difference with respect to PL is that a field in the *Y* direction (but not in the other ones, see Fig. 3A) breaks the symmetry of the PES, making it a non-degenerate double-well potential^53^ with defined reactant and product in an exothermic rearrangement (up to −6.3 kJ mol^−1^ with our strongest field). Therefore, setting the EEF in the *X* and *Z* directions or in the absence of any field, the reaction will be tunnelling back and forth with a specific frequency (that can be tuned with the strength of the field).

If the EEF is in *Y*, there will only be one possible rearrangement to the more stable state, especially at extremely cold temperatures where the higher energy reactant is unattainable (with PL it would also be possible to break the symmetry if the field is directed diagonally). This is similar to the effect experimentally obtained by carrying out asymmetric deuteration of SBV,^58,59^ which broke the degeneracy by generating slightly different zero-point energies. At a maximum field the rate will be much faster than without the field (*k*_*Y*+7.7_ = 2.1 × 10^−3^ s^−1^*vs. k*_0_ = 8.2 × 10^−6^ s^−1^), corresponding with a lower Δ*E*^‡^ without significant geometric distortions.

However, as previously proven analytically for SBV in small EEFs^53^ and experimentally for NH_3_,^54^ the most interesting part is that upon turning off the EEF in *Y* the back and forth tunnelling frequency is re-established, and inverting the field will make SBV tunnel in the opposite direction. The EEF acts here as a tunnelling switch with three positions: forward, backward, and oscillating. The influence of the EEF has similarities with the effect produced by individual atoms or even of the matrix experimentally and computationally observed on SBV and other molecules.^49,58,61,66,92,93^ Small electrostatic effects at very short distance, along other effects, can perturb both the kinetics and the thermodynamics in such sensitive reactions.

### Diazabicyclohexadiene (DBH)

DBH is a hypothetical molecule that would have a very short lifespan, if it would be possible to synthesize at all. Even if taken to the absolute zero where it should be stable (as any other system with a kinetic barrier), it was speculated that it will decompose by a [2 + 2] cycloreversion (similar to a retro-Diels–Alder, Fig. 1C)^50,64^ through what was called “Quantum Tunnelling Instability” (QTI^50,62^). This reaction, opposed to the previous ones, is highly exothermic (81 kJ mol^−1^), and therefore always one-directional. The barrier is substantial (43 kJ mol^−1^), which would make DBH absolutely stable below 100 K, if QT could be neglected.

**Fig. 4:**
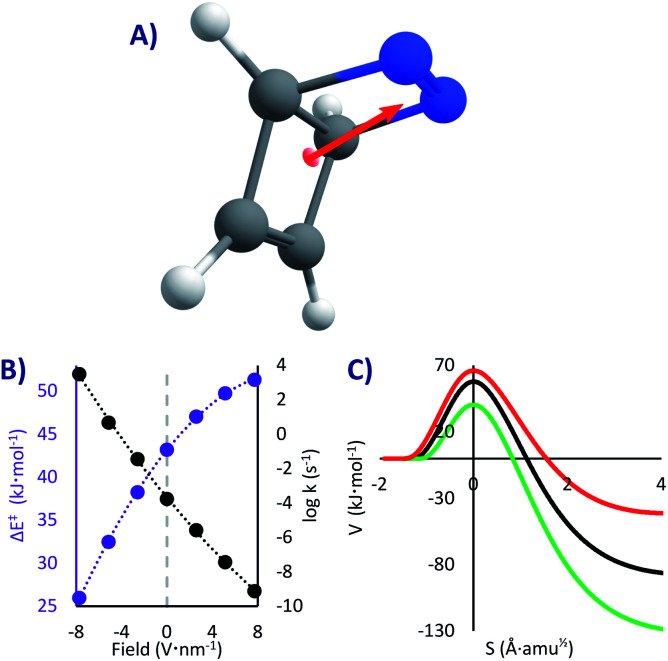
(A) DBH and its dipole moment (the EEF was set parallel to it). (B) Δ*E*^‡^ (in purple) and Tunnelling rate constants from the ground state (in black) as a function of the field strength (the grey line corresponds to the absence of EEF). (C) Potential energies at the M06-2x/6-311G(d) level *vs.* the reaction mass-scaled coordinates; in black the profile without EEF, red with a positive and green with a negative EEF of 7.7 V nm^−1^.

In this system we only studied the EEF in a single direction, parallel to the dipole moment (Fig. 4A), where the effect would be the strongest.^54^ Without an EEF, our computations predict that the half-life of DBH will be circa one hour in cryogenic conditions (*k*_0_ = 1.8 × 10^−4^ s^−1^, see Table 2), indicating that if the molecule would be synthesisable, its experimental characterization should be done extremely fast. This molecule indeed suffers from QTI.

**Table 2:** Field strength (V nm^−1^), energies of activation and reaction (kJ mol^−1^), QT rate constants from the ground state (*i.e.* approaching the absolute zero, in s^−1^), and half-lives of DBH

EEF	Δ*E*^‡^	Δ*E*_r_	*k*	*t*_1/2_
−7.7	26.0	127.7	3210	220 μs
−5.1	32.5	112.4	4.9	140 ms
−2.6	38.3	97.0	3.6 × 10^−2^	19 s
0.0	43.2	81.2	1.8 × 10^−4^	66 min
2.6	47.0	65.4	2.8 × 10^−6^	70 h
5.1	49.8	49.6	3.7 × 10^−8^	220 d
7.7	51.3	33.7	8.0 × 10^−10^	28 years

However, as can be seen in Fig. 4B and Table 2, strong electric fields (EEF of 7.7 V nm^−1^) can increase the activation energy by up to 8.1 kJ mol^−1^. This is caused by a decrease of the dipole moment through the reaction (from 3.4 D in the reactant to 2.8 D in the transition state), which leads to a higher stabilization of the intact molecule. With the maximum applied electric field, we saw a stabilization of the molecule of more than five orders of magnitude (*t*_1/2_ = 28 years, *k*_7.7_ = 8.0 × 10^−10^ s^−1^). As such, we can suggest that one conceivable way of creating and keeping molecules of this sort (and thus conquering the QTI effect) is by turning on the electricity in a nano-capacitor. Even at liquid N_2_ conditions this could be achieved (*t*_1/2_^78K^ = 21 years with a 7.7 V nm^−1^ field, compared to *t*_1/2_^78K^ = 1 h without a field). On the contrary, inverting the voltage in the nano-capacitor can condemn the molecule to instantaneously decompose, reaching *t*_1/2_^0K^ = 220 μs with a −7.7 V nm^−1^ field (this would require an immobilized system, to avoid the rotation of the molecule). Therefore, it is possible, at least in theory, to keep or destroy a molecule with the flick of a switch.

Obviously the described effect should be viable also without considering QT, as the activation energy also shapes the classical mechanism (previously observed in Diels–Alder reactions^2,4,7,8,16,17^). It would be interesting to compare the reactivity change with and without tunnelling. However, the comparison must be done on an equal footing, *i.e.* at a temperature where thermal (CVT) and tunnelling (SCT) mechanisms have similar rates (we will consider 125 K for the CVT rates, see SI for all the values). The results show that switching on the field at its maximum produces an effect a hundred times stronger with tunnelling. As explained before, QT is less sensitive to the barrier height than pure transition state theory; however, a change in the barrier height brings along a change in the most important aspect, the barrier width,^50^ as can be seen in Fig. 4C (contrary to the PL case, in most situations lower *E*_a_ leads to narrower barriers). As a result, QT tends to be altered more than a classical mechanism.

## Conclusions

The prospects of oriented external electric fields in chemical reactivity have already been established, and experimental results are slowly starting to appear.^2,4^ Up to now all the studied examples, *in vitro* or *in silico*, are only of academic significance, but the novelty and striking results make them potentially revolutionary.^9^ In this work we opened the EEF discipline to cryogenic conditions, the realm of quantum mechanical tunnelling. By selecting three prototypical cases of heavy atom tunnelling (pentalene -PL-, semibullvalene -SBV-, and diazabicyclohexadiene -DBH-) we showed the similarities and differences of the EEF effect at classical and QT regimes, which can be summarized as:

• Similar to thermal reactions, tunnelling based reactions can be tuned by oriented external electric fields.^53,55,56^ Both the strength and the direction of the field influence the outcome, which can be a speed up or down of the reaction's kinetics.

• The change in the activation energy should have a smaller effect on tunnelling compared to thermal reactions. However, the field not only affects the barrier height, but also the barrier width.

• Indeed, geometric distortions play a key role in the QT rate constants of PL. A field perpendicular to the molecular plane can force a convex geometry that, by a kind of breathing movement, augments the trajectory of the atoms. This outcome, irrelevant to thermal reactions, is the main factor in the severe slowing-down of the π bond-shifting reaction with stronger fields, even when the barrier is actually lower. This can be considered as a weird case of an inverse Arrhenius trend.

• Contrasting with the previous point, in most cases a lower barrier comes escorted by a narrower barrier (a corollary of Hammond's principle^50^). In the case of the cycloreversion of DBH, we saw that an EEF that lowers the Δ*E*^‡^ can produce a significantly faster rate on a QT mechanism compared to a classical mechanism, mostly due to the thinner barrier.

• In symmetrical reactions (*e.g.* with a degenerate double well potential as in PL and SBV) the field may break the degeneracy.^53,54^ In that case, due to the low temperatures the reaction can only tunnel one-way. However, turning of the field will reactivate the back and forth QT process. If the field does not break the symmetry, it can still serve to fine-tune the reaction frequency.

• The EEF on the volatile DBH theoretical system provides an example on how to destroy or to keep “alive” a molecule at the flick of a switch.

We acknowledge that some of these molecules are not standard systems in the organic chemistry toolbox, that some of the results are extreme-case scenarios, and that the sensibility of the kinetics to the field direction makes the accurate prediction of viable experimental rates a very challenging objective. Nevertheless, they serve us as academic examples of a novel type of reactivity. We hope that the proofs of principle and theoretical predictions provided here will stimulate the development of novel experimental tests. We believe that oriented external electric fields at cryogenic conditions, the realm of heavy atom quantum tunnelling, might one day be exploited to obtain unexpected chemical reactivity.

## Conflicts of interest

There are no conflicts to declare.

## Supplementary Material

SC-012-D0SC06295B-s001
