# Supplementary material for: Switch chemistry at cryogenic conditions: quantum tunnelling under electric fields
Source: Chem Sci. 2020 Dec 15;12(9):3179–87. doi: 10.1039/d0sc06295b (PMC8179409; doi:10.1039/d0sc06295b)
Supplement: SC-012-D0SC06295B-s001 [file SC-012-D0SC06295B-s001.pdf]

Electronic Supplementary Material (ESI) for Chemical Science.
This journal is © The Royal Society of Chemistry 2020

Electronic Supplementary Information

for

Switch Chemistry at Cryogenic Conditions: Quantum Tunnelling under Electric Fields

Omer Kirshenboim, Alexander Frenklah and Sebastian Kozuch

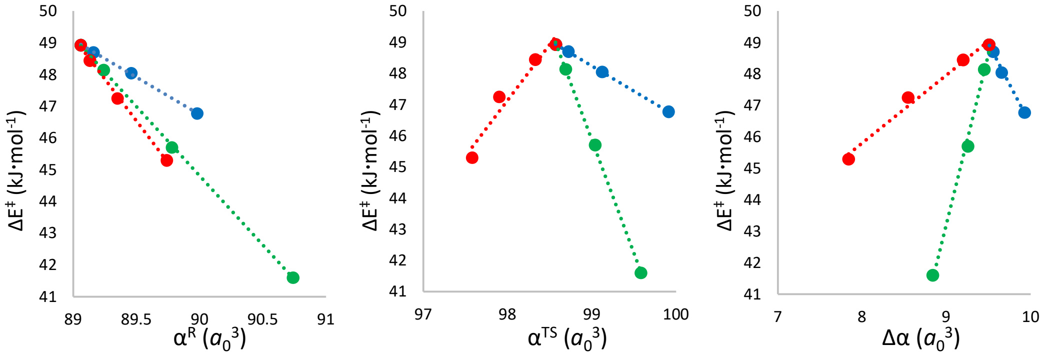

Figure S1. Activation energies vs. polarizabilities of PL in its reactant state (left), transition state (center) and the difference between them. Blue for the field in X, green in Y and red in Z.

Complete set of POLYRATE. fu15 output files including CVT, and SCT rate constants at different temperatures (in s^{-1})

**SVB**	no field			X,0.005 au			X,0.01 au			X,0.015 au	
T(K)	CVT	CVT/SCT	T(K)	CVT	CVT/SCT	T(K)	CVT	CVT/SCT	T(K)	CVT	CVT/SCT
5	1.28-293	8.15E-06	5	7.23-288	3.59E-05	5	8.02-272	1.37E-04	5	3.85-244	1.52E-03
6	6.89-243	8.15E-06	6	4.28-238	3.59E-05	6	1.00-224	1.37E-04	6	1.18-201	1.52E-03
8	1.89-179	8.15E-06	8	7.44-176	3.59E-05	8	7.95-166	1.37E-04	8	1.59-148	1.52E-03
10	2.30-141	8.15E-06	10	1.73-138	3.59E-05	10	1.82-130	1.37E-04	10	1.27-116	1.52E-03
20	4.37E-65	8.15E-06	20	1.20E-63	3.59E-05	20	1.23E-59	1.37E-04	20	1.03E-52	1.52E-03
30	1.39E-39	8.15E-06	30	1.26E-38	3.59E-05	30	5.96E-36	1.37E-04	30	2.45E-31	1.52E-03
40	8.50E-27	8.20E-06	40	4.45E-26	3.61E-05	40	4.52E-24	1.38E-04	40	1.31E-20	1.53E-03
50	4.21E-19	8.64E-06	50	1.58E-18	3.78E-05	50	6.37E-17	1.45E-04	50	3.75E-14	1.61E-03
75	8.52E-09	2.97E-05	75	2.06E-08	1.09E-04	75	2.42E-07	4.52E-04	75	1.70E-05	5.94E-03
77.36	3.63E-08	4.04E-05	77.36	8.56E-08	1.42E-04	77.36	9.33E-07	6.03E-04	77.36	5.76E-05	8.36E-03
100	1.32E-03	1.07E-02	100	2.55E-03	1.81E-02	100	1.62E-02	1.11E-01	100	3.94E-01	1.62E+00
125	1.80E+00	6.27E+00	125	3.05E+00	6.19E+00	125	1.34E+01	3.55E+01	125	1.73E+02	2.98E+02
150	2.28E+02	5.93E+02	150	3.54E+02	4.67E+02	150	1.22E+03	2.22E+03	150	1.03E+04	1.28E+04
175	7.39E+03	1.64E+04	175	1.08E+04	1.18E+04	175	3.12E+04	4.65E+04	175	1.94E+05	2.10E+05
194.7	6.18E+04	1.26E+05	194.7	8.70E+04	8.85E+04	194.7	2.26E+05	3.05E+05	194.7	1.17E+06	1.19E+06
200	1.02E+05	2.03E+05	200	1.42E+05	1.43E+05	200	3.60E+05	4.76E+05	200	1.79E+06	1.80E+06
225	7.96E+05	1.46E+06	225	1.07E+06	1.03E+06	225	2.45E+06	2.99E+06	225	1.02E+07	9.88E+06
250	4.15E+06	7.16E+06	250	5.41E+06	5.07E+06	250	1.14E+07	1.32E+07	250	4.14E+07	3.93E+07
273.15	1.47E+07	2.42E+07	273.15	1.88E+07	1.74E+07	273.15	3.72E+07	4.16E+07	273.15	1.21E+08	1.14E+08
275	1.61E+07	2.65E+07	275	2.05E+07	1.90E+07	275	4.05E+07	4.52E+07	275	1.31E+08	1.23E+08
298.15	4.64E+07	7.34E+07	298.15	5.81E+07	5.34E+07	298.15	1.09E+08	1.18E+08	298.15	3.22E+08	3.00E+08
300	5.02E+07	7.91E+07	300	6.27E+07	5.76E+07	300	1.17E+08	1.27E+08	300	3.44E+08	3.21E+08
325	1.32E+08	2.01E+08	325	1.62E+08	1.48E+08	325	2.88E+08	3.07E+08	325	7.82E+08	7.27E+08
350	3.02E+08	4.47E+08	350	3.66E+08	3.35E+08	350	6.26E+08	6.57E+08	350	1.59E+09	1.47E+09
373.15	5.92E+08	8.55E+08	373.15	7.08E+08	6.48E+08	373.15	1.17E+09	1.22E+09	373.15	2.81E+09	2.61E+09
375	6.23E+08	8.97E+08	375	7.44E+08	6.81E+08	375	1.23E+09	1.28E+09	375	2.93E+09	2.72E+09
400	1.17E+09	1.65E+09	400	1.39E+09	1.27E+09	400	2.22E+09	2.29E+09	400	5.04E+09	4.68E+09

SVB	Y,0.005 au			Y,0.01 au			Y,0.015 au	
T(K)	CVT	CVT/SCT	T(K)	CVT	CVT/SCT	T(K)	CVT	CVT/SCT
5	2.31-280	1.09E-04	5	1.47-265	1.20E-03	5	1.35-248	2.05E-03
6	7.68-232	1.09E-04	6	1.67-219	1.20E-03	6	2.27-205	2.05E-03
8	3.65-171	1.09E-04	8	6.53-162	1.20E-03	8	2.60-151	2.05E-03
10	9.79-135	1.09E-04	10	2.47-127	1.20E-03	10	7.47-119	2.05E-03
20	9.00E-62	1.09E-04	20	4.52E-58	1.20E-03	20	7.87E-54	2.05E-03
30	2.25E-37	1.09E-04	30	6.59E-35	1.20E-03	30	4.42E-32	2.05E-03
40	3.86E-25	1.09E-04	40	2.74E-23	1.21E-03	40	3.61E-21	2.06E-03
50	8.91E-18	1.14E-04	50	2.69E-16	1.25E-03	50	1.34E-14	2.15E-03
75	6.52E-08	3.02E-04	75	6.33E-07	2.93E-03	75	8.56E-06	5.82E-03
77.36	2.61E-07	3.86E-04	77.36	2.37E-06	3.64E-03	77.36	2.96E-05	7.54E-03
100	6.06E-03	4.63E-02	100	3.33E-02	3.09E-01	100	2.35E-01	8.40E-01
125	6.09E+00	1.48E+01	125	2.38E+01	8.18E+01	125	1.14E+02	1.65E+02
150	6.30E+02	1.00E+03	150	1.97E+03	4.69E+03	150	7.22E+03	7.94E+03
175	1.77E+04	2.29E+04	175	4.70E+04	9.15E+04	175	1.43E+05	1.41E+05
194.7	1.36E+05	1.59E+05	194.7	3.26E+05	5.71E+05	194.7	8.87E+05	8.40E+05
200	2.19E+05	2.53E+05	200	5.16E+05	8.81E+05	200	1.36E+06	1.28E+06
225	1.57E+06	1.69E+06	225	3.36E+06	5.24E+06	225	7.97E+06	7.34E+06
250	7.65E+06	7.91E+06	250	1.52E+07	2.21E+07	250	3.30E+07	3.01E+07
273.15	2.58E+07	2.60E+07	273.15	4.84E+07	6.70E+07	273.15	9.81E+07	8.92E+07
275	2.81E+07	2.83E+07	275	5.26E+07	7.27E+07	275	1.06E+08	9.66E+07
298.15	7.77E+07	7.70E+07	298.15	1.39E+08	1.84E+08	298.15	2.64E+08	2.40E+08
300	8.37E+07	8.28E+07	300	1.49E+08	1.97E+08	300	2.83E+08	2.57E+08
325	2.11E+08	2.07E+08	325	3.60E+08	4.62E+08	325	6.49E+08	5.91E+08
350	4.69E+08	4.55E+08	350	7.70E+08	9.63E+08	350	1.33E+09	1.21E+09
373.15	8.95E+08	8.64E+08	373.15	1.43E+09	1.75E+09	373.15	2.37E+09	2.17E+09
375	9.39E+08	9.06E+08	375	1.49E+09	1.83E+09	375	2.48E+09	2.27E+09
400	1.73E+09	1.66E+09	400	2.67E+09	3.21E+09	400	4.29E+09	3.93E+09

**SVB**	Z,0.005 au			Z,0.01 au			Z,0.015 au	
T(K)	CVT	CVT/SCT	T(K)	CVT	CVT/SCT	T(K)	CVT	CVT/SCT
5	1.52-293	1.81E-05	5	2.47-293	1.44E-05	5	1.40-292	4.78E-06
6	7.93-243	1.81E-05	6	1.19-242	1.44E-05	6	5.06-242	4.78E-06
8	2.10-179	1.81E-05	8	2.86-179	1.44E-05	8	8.45-179	4.78E-06
10	2.51-141	1.81E-05	10	3.20-141	1.44E-05	10	7.63-141	4.78E-06
20	4.56E-65	1.81E-05	20	5.15E-65	1.44E-05	20	7.96E-65	4.78E-06
30	1.43E-39	1.81E-05	30	1.55E-39	1.44E-05	30	2.07E-39	4.78E-06
40	8.69E-27	1.82E-05	40	9.25E-27	1.44E-05	40	1.15E-26	4.81E-06
50	4.28E-19	1.90E-05	50	4.50E-19	1.51E-05	50	5.36E-19	5.09E-06
75	8.62E-09	5.50E-05	75	8.92E-09	4.53E-05	75	1.00E-08	1.92E-05
77.36	3.68E-08	7.17E-05	77.36	3.80E-08	5.95E-05	77.36	4.26E-08	2.68E-05
100	1.33E-03	1.24E-02	100	1.36E-03	8.92E-03	100	1.49E-03	7.32E-03
125	1.81E+00	6.60E+00	125	1.85E+00	3.39E+00	125	1.99E+00	3.28E+00
150	2.29E+02	6.13E+02	150	2.34E+02	2.83E+02	150	2.48E+02	2.87E+02
175	7.45E+03	1.68E+04	175	7.58E+03	7.76E+03	175	7.99E+03	8.01E+03
194.7	6.23E+04	1.29E+05	194.7	6.33E+04	6.10E+04	194.7	6.65E+04	6.33E+04
200	1.03E+05	2.08E+05	200	1.04E+05	9.95E+04	200	1.10E+05	1.03E+05
225	8.01E+05	1.50E+06	225	8.13E+05	7.49E+05	225	8.49E+05	7.79E+05
250	4.17E+06	7.33E+06	250	4.23E+06	3.84E+06	250	4.41E+06	3.99E+06
273.15	1.48E+07	2.48E+07	273.15	1.50E+07	1.35E+07	273.15	1.56E+07	1.40E+07
275	1.62E+07	2.71E+07	275	1.64E+07	1.48E+07	275	1.71E+07	1.54E+07
298.15	4.67E+07	7.50E+07	298.15	4.73E+07	4.25E+07	298.15	4.90E+07	4.42E+07
300	5.04E+07	8.08E+07	300	5.11E+07	4.60E+07	300	5.30E+07	4.77E+07
325	1.32E+08	2.05E+08	325	1.34E+08	1.21E+08	325	1.39E+08	1.25E+08
350	3.03E+08	4.56E+08	350	3.07E+08	2.77E+08	350	3.18E+08	2.87E+08
373.15	5.94E+08	8.70E+08	373.15	6.02E+08	5.43E+08	373.15	6.21E+08	5.62E+08
375	6.25E+08	9.13E+08	375	6.33E+08	5.71E+08	375	6.53E+08	5.91E+08
400	1.18E+09	1.68E+09	400	1.19E+09	1.08E+09	400	1.23E+09	1.12E+09

**PL**	no field			X, 0.005 au			X,0.01 au			X,0.015 au	
T(K)	CVT	CVT/SCT	T(K)	CVT	CVT/SCT	T(K)	CVT	CVT/SCT	T(K)	CVT	CVT/SCT
8	7.06-259	1.09E+08	8	2.00-257	1.06E+08	8	1.86-253	1.14E+08	8	1.45-245	1.32E+08
10	6.59-205	1.09E+08	10	9.57-204	1.06E+08	10	1.43-200	1.14E+08	10	2.95-194	1.32E+08
20	7.36E-97	1.09E+08	20	2.80E-96	1.06E+08	20	1.09E-94	1.14E+08	20	1.56E-91	1.32E+08
30	9.18E-61	1.10E+08	30	2.24E-60	1.08E+08	30	2.57E-59	1.16E+08	30	3.27E-57	1.34E+08
40	1.13E-42	1.13E+08	40	2.22E-42	1.11E+08	40	1.38E-41	1.20E+08	40	5.25E-40	1.39E+08
50	8.67E-32	1.19E+08	50	1.48E-31	1.17E+08	50	6.43E-31	1.26E+08	50	1.18E-29	1.46E+08
75	3.24E-17	1.41E+08	75	4.65E-17	1.39E+08	75	1.24E-16	1.51E+08	75	8.66E-16	1.76E+08
77.36	2.52E-16	1.44E+08	77.36	3.58E-16	1.42E+08	77.36	9.26E-16	1.54E+08	77.36	6.10E-15	1.79E+08
100	6.97E-10	1.76E+08	100	9.16E-10	1.74E+08	100	1.92E-09	1.88E+08	100	8.26E-09	2.20E+08
125	1.86E-05	2.24E+08	125	2.32E-05	2.22E+08	125	4.19E-05	2.41E+08	125	1.35E-04	2.82E+08
150	1.72E-02	2.87E+08	150	2.07E-02	2.87E+08	150	3.40E-02	3.11E+08	150	9.03E-02	3.65E+08
175	2.32E+00	3.73E+08	175	2.73E+00	3.73E+08	175	4.17E+00	4.06E+08	175	9.65E+00	4.77E+08
194.7	4.62E+01	4.60E+08	194.7	5.34E+01	4.62E+08	194.7	7.84E+01	5.03E+08	194.7	1.67E+02	5.92E+08
200	9.35E+01	4.87E+08	200	1.08E+02	4.90E+08	200	1.57E+02	5.34E+08	200	3.26E+02	6.28E+08
225	1.68E+03	6.41E+08	225	1.91E+03	6.48E+08	225	2.66E+03	7.06E+08	225	5.11E+03	8.31E+08
250	1.71E+04	8.48E+08	250	1.91E+04	8.61E+08	250	2.59E+04	9.39E+08	250	4.66E+04	1.11E+09
273.15	1.01E+05	1.10E+09	273.15	1.12E+05	1.13E+09	273.15	1.48E+05	1.23E+09	273.15	2.53E+05	1.45E+09
275	1.15E+05	1.13E+09	275	1.27E+05	1.15E+09	275	1.68E+05	1.26E+09	275	2.86E+05	1.48E+09
298.15	5.05E+05	1.48E+09	298.15	5.57E+05	1.51E+09	298.15	7.18E+05	1.65E+09	298.15	1.18E+06	1.95E+09
300	5.63E+05	1.51E+09	300	6.21E+05	1.55E+09	300	7.99E+05	1.69E+09	300	1.31E+06	1.99E+09
325	2.18E+06	2.02E+09	325	2.38E+06	2.08E+09	325	3.01E+06	2.27E+09	325	4.74E+06	2.68E+09
350	6.96E+06	2.70E+09	350	7.57E+06	2.79E+09	350	9.41E+06	3.05E+09	350	1.43E+07	3.59E+09
373.15	1.78E+07	3.53E+09	373.15	1.93E+07	3.66E+09	373.15	2.37E+07	3.99E+09	373.15	3.51E+07	4.70E+09
375	1.91E+07	3.61E+09	375	2.07E+07	3.74E+09	375	2.54E+07	4.08E+09	375	3.76E+07	4.80E+09
400	4.63E+07	4.79E+09	400	5.00E+07	4.97E+09	400	6.05E+07	5.43E+09	400	8.74E+07	6.38E+09

**PL**	Y,0.005 au			Y,0.01 au			Y,0.015 au	
T(K)	CVT	CVT/SCT	T(K)	CVT	CVT/SCT	T(K)	CVT	CVT/SCT
8	1.08-253	1.14E+08	8	5.86-238	1.58E+08	8	1.81-211	2.88E+08
10	9.29-201	1.14E+08	10	3.58-188	1.58E+08	10	5.57-167	2.88E+08
20	8.74E-95	1.15E+08	20	1.72E-88	1.58E+08	20	6.77E-78	2.88E+08
30	2.22E-59	1.16E+08	30	3.47E-55	1.60E+08	30	4.01E-48	2.90E+08
40	1.23E-41	1.19E+08	40	1.72E-38	1.64E+08	40	3.39E-33	2.96E+08
50	5.85E-31	1.25E+08	50	1.91E-28	1.71E+08	50	3.26E-24	3.08E+08
75	1.15E-16	1.49E+08	75	5.41E-15	2.02E+08	75	3.54E-12	3.60E+08
77.36	8.63E-16	1.52E+08	77.36	3.59E-14	2.06E+08	77.36	1.93E-11	3.66E+08
100	1.80E-09	1.85E+08	100	3.20E-08	2.51E+08	100	4.08E-06	4.43E+08
125	3.97E-05	2.36E+08	125	3.92E-04	3.19E+08	125	1.87E-02	5.62E+08
150	3.23E-02	3.05E+08	150	2.17E-01	4.12E+08	150	5.38E+00	7.24E+08
175	3.98E+00	3.97E+08	175	2.02E+01	5.36E+08	175	3.15E+02	9.45E+08
194.7	7.49E+01	4.92E+08	194.7	3.21E+02	6.65E+08	194.7	3.77E+03	1.17E+09
200	1.50E+02	5.21E+08	200	6.17E+02	7.05E+08	200	6.77E+03	1.25E+09
225	2.55E+03	6.89E+08	225	8.93E+03	9.34E+08	225	7.47E+04	1.65E+09
250	2.48E+04	9.17E+08	250	7.65E+04	1.24E+09	250	5.15E+05	2.21E+09
273.15	1.42E+05	1.20E+09	273.15	3.96E+05	1.63E+09	273.15	2.26E+06	2.90E+09
275	1.61E+05	1.23E+09	275	4.47E+05	1.67E+09	275	2.52E+06	2.97E+09
298.15	6.91E+05	1.61E+09	298.15	1.77E+06	2.19E+09	298.15	8.67E+06	3.90E+09
300	7.69E+05	1.65E+09	300	1.95E+06	2.24E+09	300	9.49E+06	3.99E+09
325	2.90E+06	2.21E+09	325	6.84E+06	3.01E+09	325	2.93E+07	5.36E+09
350	9.08E+06	2.97E+09	350	2.01E+07	4.05E+09	350	7.74E+07	7.16E+09
373.15	2.29E+07	3.90E+09	373.15	4.80E+07	5.29E+09	373.15	1.70E+08	9.32E+09
375	2.45E+07	3.98E+09	375	5.13E+07	5.41E+09	375	1.80E+08	9.51E+09
400	5.85E+07	5.30E+09	400	1.17E+08	7.18E+09	400	3.78E+08	1.25E+10

**PL**	Z, 0.005 au			Z,0.01 au			Z,0.015 au	
T(K)	CVT	CVT/SCT	T(K)	CVT	CVT/SCT	T(K)	CVT	CVT/SCT
8	9.68-257	2.47E+07	8	2.23-251	3.61E+05	8	8.79-244	1.04E+03
10	3.37-203	2.47E+07	10	6.57-199	3.61E+05	10	7.83-193	1.04E+03
20	5.26E-96	2.47E+07	20	7.30E-94	3.61E+05	20	7.95E-91	1.04E+03
30	3.39E-60	2.50E+07	30	9.04E-59	3.65E+05	30	9.53E-57	1.07E+03
40	3.00E-42	2.57E+07	40	3.49E-41	3.85E+05	40	1.14E-39	1.18E+03
50	1.87E-31	2.70E+07	50	1.32E-30	4.30E+05	50	2.13E-29	1.47E+03
75	5.30E-17	3.31E+07	75	1.90E-16	7.01E+05	75	1.19E-15	4.17E+03
77.36	4.05E-16	3.38E+07	77.36	1.39E-15	7.40E+05	77.36	8.21E-15	4.70E+03
100	9.89E-10	4.33E+07	100	2.51E-09	1.30E+06	100	9.70E-09	1.54E+04
125	2.41E-05	5.88E+07	125	4.97E-05	2.47E+06	125	1.43E-04	5.49E+04
150	2.11E-02	8.15E+07	150	3.76E-02	4.70E+06	150	8.89E-02	1.79E+05
175	2.72E+00	1.14E+08	175	4.38E+00	8.85E+06	175	8.97E+00	5.35E+05
194.7	5.27E+01	1.50E+08	194.7	7.96E+01	1.44E+07	194.7	1.49E+02	1.20E+06
200	1.06E+02	1.62E+08	200	1.58E+02	1.64E+07	200	2.90E+02	1.48E+06
225	1.85E+03	2.31E+08	225	2.59E+03	2.99E+07	225	4.36E+03	3.79E+06
250	1.84E+04	3.30E+08	250	2.45E+04	5.36E+07	250	3.85E+04	9.08E+06
273.15	1.07E+05	4.62E+08	273.15	1.37E+05	9.01E+07	273.15	2.03E+05	1.92E+07
275	1.22E+05	4.74E+08	275	1.55E+05	9.38E+07	275	2.30E+05	2.04E+07
298.15	5.28E+05	6.62E+08	298.15	6.52E+05	1.54E+08	298.15	9.22E+05	4.07E+07
300	5.89E+05	6.80E+08	300	7.25E+05	1.61E+08	300	1.02E+06	4.29E+07
325	2.24E+06	9.73E+08	325	2.68E+06	2.68E+08	325	3.62E+06	8.52E+07
350	7.09E+06	1.38E+09	350	8.26E+06	4.36E+08	350	1.07E+07	1.60E+08
373.15	1.80E+07	1.90E+09	373.15	2.05E+07	6.67E+08	373.15	2.58E+07	2.74E+08
375	1.93E+07	1.95E+09	375	2.19E+07	6.89E+08	375	2.76E+07	2.86E+08
400	4.64E+07	2.72E+09	400	5.17E+07	1.06E+09	400	6.31E+07	4.87E+08

**CBD**	no field			X,-0.005 au			X,-0.01 au			X,-0.015 au	
T(K)	CVT	CVT/SCT	T(K)	CVT	CVT/SCT	T(K)	CVT	CVT/SCT	T(K)	CVT	CVT/SCT
8	6.67-273	1.76E-04	8	2.56-240	3.62E-02	8	4.83-203	4.91E+00	8	7.41-161	3.23E+03
10	4.10-216	1.76E-04	10	4.77-190	3.61E-02	10	3.15-160	4.97E+00	10	1.77-126	3.25E+03
20	1.98-102	1.76E-04	20	2.13E-89	3.61E-02	20	1.72E-74	5.09E+00	20	1.29E-57	3.27E+03
30	1.85E-64	1.76E-04	30	9.00E-56	3.61E-02	30	7.79E-46	5.13E+00	30	1.38E-34	3.28E+03
40	1.95E-45	1.76E-04	40	6.37E-39	3.61E-02	40	1.80E-31	5.15E+00	40	4.91E-23	3.29E+03
50	5.31E-34	1.76E-04	50	8.63E-29	3.63E-02	50	7.89E-23	5.18E+00	50	4.41E-16	3.30E+03
75	1.05E-18	1.98E-04	75	3.13E-15	3.98E-02	75	2.95E-11	5.68E+00	75	9.27E-07	3.61E+03
77.36	9.06E-18	2.03E-04	77.36	2.11E-14	4.07E-02	77.36	1.50E-10	5.80E+00	77.36	3.45E-06	3.68E+03
100	5.20E-11	3.51E-04	100	2.09E-08	6.29E-02	100	2.00E-05	8.64E+00	100	4.72E-02	5.26E+03
125	2.30E-06	2.00E-03	125	2.79E-04	2.41E-01	125	6.75E-02	2.83E+01	125	3.39E+01	1.36E+04
150	3.04E-03	6.78E-02	150	1.65E-01	4.00E+00	150	1.60E+01	2.90E+02	150	2.86E+03	7.45E+04
175	5.35E-01	3.37E+00	175	1.64E+01	1.11E+02	175	8.29E+02	4.22E+03	175	7.06E+04	5.56E+05
194.7	1.26E+01	4.92E+01	194.7	2.74E+02	1.16E+03	194.7	9.30E+03	2.82E+04	194.7	5.06E+05	2.43E+06
200	2.67E+01	9.46E+01	200	5.34E+02	2.06E+03	200	1.65E+04	4.52E+04	200	8.06E+05	3.51E+06
225	5.72E+02	1.46E+03	225	8.19E+03	2.31E+04	225	1.73E+05	3.32E+05	225	5.48E+06	1.69E+07
250	6.77E+03	1.40E+04	250	7.42E+04	1.71E+05	250	1.15E+06	1.78E+06	250	2.59E+07	6.37E+07
273.15	4.52E+04	8.16E+04	273.15	4.04E+05	8.18E+05	273.15	4.97E+06	6.72E+06	273.15	8.58E+07	1.82E+08
275	5.19E+04	9.29E+04	275	4.58E+05	9.17E+05	275	5.53E+06	7.42E+06	275	9.36E+07	1.96E+08
298.15	2.55E+05	4.14E+05	298.15	1.90E+06	3.46E+06	298.15	1.89E+07	2.31E+07	298.15	2.57E+08	4.82E+08
300	2.87E+05	4.62E+05	300	2.11E+06	3.81E+06	300	2.07E+07	2.52E+07	300	2.76E+08	5.15E+08
325	1.23E+06	1.83E+06	325	7.77E+06	1.30E+07	325	6.38E+07	7.26E+07	325	6.98E+08	1.19E+09
350	4.34E+06	6.05E+06	350	2.39E+07	3.75E+07	350	1.69E+08	1.83E+08	350	1.55E+09	2.46E+09
373.15	1.20E+07	1.60E+07	373.15	5.97E+07	8.91E+07	373.15	3.72E+08	3.91E+08	373.15	2.98E+09	4.48E+09
375	1.30E+07	1.73E+07	375	6.39E+07	9.51E+07	375	3.95E+08	4.14E+08	375	3.13E+09	4.69E+09
400	3.41E+07	4.36E+07	400	1.52E+08	2.17E+08	400	8.35E+08	8.53E+08	400	5.81E+09	8.30E+09

CBD	X,0.005 au			X, 0.01 au			X, 0.015 au	
T(K)	CVT	CVT/SCT	T(K)	CVT	CVT/SCT	T(K)	CVT	CVT/SCT
10	1.12-235	2.75E-06	10	4.56-250	3.69E-08	10	4.81-258	7.95E-10
20	3.29-112	2.75E-06	20	2.10-119	3.69E-08	20	2.17-123	7.95E-10
30	5.61E-71	2.75E-06	30	8.99E-76	3.69E-08	30	1.98E-78	7.95E-10
40	2.52E-50	2.75E-06	40	6.40E-54	3.69E-08	40	6.52E-56	7.96E-10
50	6.54E-38	2.76E-06	50	8.70E-41	3.72E-08	50	2.22E-42	8.02E-10
75	2.61E-21	3.14E-06	75	3.17E-23	4.40E-08	75	2.76E-24	1.00E-09
77.36	2.70E-20	3.24E-06	77.36	3.74E-22	4.58E-08	77.36	3.51E-23	1.06E-09
100	5.79E-13	6.10E-06	100	2.12E-14	1.08E-07	100	3.41E-15	3.47E-09
125	6.32E-08	5.01E-05	125	4.50E-09	1.87E-06	125	1.05E-09	1.68E-07
150	1.52E-04	3.11E-03	150	1.69E-05	2.75E-04	150	5.04E-06	5.59E-05
175	4.12E-02	2.51E-01	175	6.28E-03	3.63E-02	175	2.24E-03	1.00E-02
194.7	1.26E+00	4.89E+00	194.7	2.34E-01	9.17E-01	194.7	9.29E-02	2.86E-01
200	2.84E+00	1.00E+01	200	5.50E-01	2.00E+00	200	2.24E-01	6.41E-01
225	7.83E+01	2.04E+02	225	1.82E+01	5.11E+01	225	8.26E+00	1.81E+01
250	1.13E+03	2.42E+03	250	3.07E+02	7.26E+02	250	1.51E+02	2.79E+02
273.15	8.84E+03	1.65E+04	273.15	2.68E+03	5.66E+03	273.15	1.41E+03	2.31E+03
275	1.03E+04	1.90E+04	275	3.14E+03	6.58E+03	275	1.66E+03	2.70E+03
298.15	5.75E+04	9.68E+04	298.15	1.93E+04	3.72E+04	298.15	1.07E+04	1.61E+04
300	6.52E+04	1.09E+05	300	2.21E+04	4.23E+04	300	1.23E+04	1.84E+04
325	3.15E+05	4.87E+05	325	1.16E+05	2.07E+05	325	6.82E+04	9.49E+04
350	1.22E+06	1.78E+06	350	4.86E+05	8.21E+05	350	2.98E+05	3.93E+05
373.15	3.69E+06	5.13E+06	373.15	1.55E+06	2.51E+06	373.15	9.83E+05	1.25E+06
375	4.00E+06	5.55E+06	375	1.69E+06	2.73E+06	375	1.08E+06	1.36E+06
400	1.13E+07	1.51E+07	400	5.08E+06	7.86E+06	400	3.33E+06	4.08E+06

Example of POLYRATE input files:

.dat

```
*GENERAL

TITLE
  Semibull_fieldX100_0.001
END

DL ISPE

ATOMS
1 C
2 C
3 C
4 C
5 C
6 C
7 C
8 C
9 H
10 H
11 H
12 H
13 H
14 H
15 H
16 H
END

NOSUPERMOL

*SECOND

HESSCAL hhook

FPRINT

*OPTIMIZATION

PRINT

OPTMIN ohook
OPTTS ohook

*REACT1
INITGEO hooks
GEOM
1
2
3
4
5
6
7
8
9
10
11
12
13
14
15
16
END
SPECIES nonlinrp

*PROD1
INITGEO hooks
GEOM
1
2
3
4
5
6
7
8
9
10
11
12
13
14
15
16
END
SPECIES nonlinrp

*START
INITGEO hooks
GEOM
1
2
3
4
5
6
7
8
9
10
11
12
13
14
15
16
END
SPECIES nonlints
PROJECT

*PATH
SYMMETRY
INTMU 3
SSTEP 0.001
RPM pagem
SRANGE
  SLP 3.
  SLM -3.
END
PRPATH
  coord 1 2
  xmol
  freq 42
END

*TUNNEL
ZCT
SCT
QRST
  harmonic
  mode 42
  states all
END

*RATE
FORWARDK
SIGMAF 1
TST
CVT
PRDELG
PRPART rtp

TEMP
4
5
6
8
10
20
30
40
50
75
77.355
100
125
150
175
194.7
200
225
250
273.15
275
298.15
300
325
350
373.15
375
400
END

ANALYSIS
4
5
6
8
10
20
30
40
50
75
77.355
100
125
150
175
194.7
200
225
250
273.15
275
298.15
300
325
350
373.15
375
400
END

EACT
6. 10.
10. 20.
20. 50.
50. 100.
200. 225.
300. 325.
END

GTLOG*
```

.51

```
*ISPEGEN
ENERXN 0.0000     # Corrected Exergonicity
ENESAD 7.515453   # Corrected Activation energy (deltaE#)
MEPTYPER one
MEPTYPEP one
RCINFO
 SRC -5.6635  # Position of the reactant in Bohr (s from fu28)
END
PCINFO
 SPC 5.6635  # Position of the product in Bohr
END
```

.70

```
*GRGENERAL
GRRESTART

*GRSTART
CHARGE 0
MULTIPLICITY 1

*GRCOMMON

GRENER
%mem=48gb
%nproc=24
#n m062x/6-311g(d)  units(au) fchk nosymm int(ultrafine) field=X+100
END

GRFIRST
%mem=48gb
%nproc=24
#n m062x/6-311g(d)  units(au) fchk nosymm force int(ultrafine) field=X+100
END

GRSEC
%mem=48gb
%nproc=24
#n m062x/6-311g(d)  units(au) fchk nosymm freq int(ultrafine) field=X+100
END
```

.71

```
%mem=48gb
%nproc=24
#n m062x/6-311g(d)  fchk  nosymm int(ultrafine) field=X+100


0 1
C    0.000328    0.727494    0.965883
C    0.802265    1.171744   -0.222409
C    1.557324   -0.004858   -0.694056
C    1.184275   -1.110751   -0.037447
C    0.003522   -0.816865    0.883473
C   -1.166420   -1.120178   -0.049295
C   -1.524982   -0.020748   -0.718084
C   -0.780118    1.158624   -0.232708
H   -0.023886    1.261038    1.907418
H    1.233153    2.165061   -0.267553
H    2.237217    0.017848   -1.539309
H    1.494090   -2.121586   -0.281176
H   -0.005973   -1.348799    1.834307
H   -1.529565   -2.123479   -0.234419
H   -2.255651    0.021630   -1.515309
H   -1.225580    2.143826   -0.281720
```

.73

```
%mem=48gb
%nproc=24
#n m062x/6-311g(d)  fchk  nosymm int(ultrafine) field=X+100


0 1
C    0.000328    0.727494    0.965883
C    0.802265    1.171744   -0.222409
C    1.557324   -0.004858   -0.694056
C    1.184275   -1.110751   -0.037447
C    0.003522   -0.816865    0.883473
C   -1.166420   -1.120178   -0.049295
C   -1.524982   -0.020748   -0.718084
C   -0.780118    1.158624   -0.232708
H   -0.023886    1.261038    1.907418
H    1.233153    2.165061   -0.267553
H    2.237217    0.017848   -1.539309
H    1.494090   -2.121586   -0.281176
H   -0.005973   -1.348799    1.834307
H   -1.529565   -2.123479   -0.234419
H   -2.255651    0.021630   -1.515309
H   -1.225580    2.143826   -0.281720
```

.75

```
%mem=48gb
%nproc=24
#n m062x/6-311g(d)  fchk  nosymm int(ultrafine) field=X+100


0 1
C    0.001711    0.775769    0.947197
C    1.012681    1.141983   -0.099987
C    1.559474   -0.000000   -0.670843
C    1.012681   -1.141983   -0.099987
C    0.001711   -0.775769    0.947197
C   -0.991538   -1.137592   -0.109963
C   -1.527610   -0.000000   -0.693069
C   -0.991538    1.137592   -0.109963
H   -0.015454    1.301609    1.898750
H    1.271574    2.164687   -0.335244
H    2.191831   -0.000000   -1.551507
H    1.271574   -2.164687   -0.335244
H   -0.015454   -1.301609    1.898750
H   -1.283061   -2.156408   -0.320407
H   -2.215517   -0.000000   -1.527679
H   -1.283061    2.156408   -0.320407
```

XYZ cartesian coordinates (species, field direction, field strength)

SBV (reac'/prod') – no field

C	1.142078	-0.416037	0.000000
C	0.396241	-1.041187	1.175523
C	-0.633666	-0.270215	1.539521
C	-0.633666	1.001882	0.790094
C	0.637507	1.045680	0.000000
C	-0.633666	1.001882	-0.790094
C	-0.633666	-0.270215	-1.539521
C	0.396241	-1.041187	-1.175523
H	2.223267	-0.550866	0.000000
H	0.577222	-2.055382	1.511996
H	-1.406689	-0.548643	2.245263
H	-1.048977	1.900415	1.229028
H	1.309200	1.894459	0.000000
H	-1.048977	1.900415	-1.229028
H	-1.406689	-0.548643	-2.245263
H	0.577222	-2.055382	-1.511996

SBV (TS) – no field

C	0.000000	0.775570	0.947697
C	0.999525	1.139596	-0.106644
C	1.539893	-0.000000	-0.684275
C	0.999525	-1.139596	-0.106644
C	-0.000000	-0.775570	0.947697
C	-0.999525	-1.139596	-0.106644
C	-1.539893	0.000000	-0.684275
C	-0.999525	1.139596	-0.106644
H	0.000000	1.302466	1.898747
H	1.275304	2.160438	-0.328480
H	2.199264	-0.000000	-1.542590
H	1.275304	-2.160438	-0.328480
H	-0.000000	-1.302466	1.898747
H	-1.275304	-2.160438	-0.328480
H	-2.199264	0.000000	-1.542590
H	-1.275304	2.160438	-0.328480

SBV (reac'/prod'), X, 50

C	0.000167	0.727370	0.966149
C	0.795799	1.168623	-0.225283
C	1.547955	-0.009022	-0.700692
C	1.180031	-1.112799	-0.040165
C	0.001773	-0.816871	0.883366
C	-1.171102	-1.117516	-0.046088
C	-1.531834	-0.016971	-0.712669
C	-0.784725	1.162056	-0.230417
H	-0.011950	1.260937	1.907857
H	1.230938	2.159553	-0.270750
H	2.240889	0.019035	-1.533748
H	1.503205	-2.122237	-0.269025
H	-0.002954	-1.348830	1.834218
H	-1.521013	-2.123159	-0.245569
H	-2.250018	0.020917	-1.521808
H	-1.227162	2.148915	-0.277782

SBV (TS), X, 50

C	0.000856	0.775618	0.948468
C	1.005430	1.140732	-0.102859
C	1.548783	0.000000	-0.677265
C	1.005430	-1.140732	-0.102859
C	0.000856	-0.775618	0.948468
C	-0.994883	-1.138556	-0.107827
C	-1.532802	0.000000	-0.688358
C	-0.994883	1.138556	-0.107827
H	-0.007730	1.302255	1.899641
H	1.272936	2.162542	-0.331102
H	2.194405	0.000000	-1.546948
H	1.272936	-2.162542	-0.331102
H	-0.007730	-1.302255	1.899641
H	-1.278662	-2.158390	-0.323747
H	-2.206278	-0.000000	-1.534983
H	-1.278662	2.158390	-0.323747

SBV (reac'/prod'), X, 100

C	0.000328	0.727494	0.965883
C	0.802265	1.171744	-0.222409
C	1.557324	-0.004858	-0.694056
C	1.184275	-1.110751	-0.037447
C	0.003522	-0.816865	0.883473
C	-1.166420	-1.120178	-0.049295
C	-1.524982	-0.020748	-0.718084
C	-0.780118	1.158624	-0.232708
H	-0.023886	1.261038	1.907418
H	1.233153	2.165061	-0.267553
H	2.237217	0.017848	-1.539309
H	1.494090	-2.121586	-0.281176
H	-0.005973	-1.348799	1.834307
H	-1.529565	-2.123479	-0.234419
H	-2.255651	0.021630	-1.515309
H	-1.225580	2.143826	-0.281720

SBV (TS), X, 100

C	0.001711	0.775769	0.947197
C	1.012681	1.141983	-0.099987
C	1.559474	-0.000000	-0.670843
C	1.012681	-1.141983	-0.099987
C	0.001711	-0.775769	0.947197
C	-0.991538	-1.137592	-0.109963
C	-1.527610	-0.000000	-0.693069
C	-0.991538	1.137592	-0.109963
H	-0.015454	1.301609	1.898750
H	1.271574	2.164687	-0.335244
H	2.191831	-0.000000	-1.551507
H	1.271574	-2.164687	-0.335244
H	-0.015454	-1.301609	1.898750
H	-1.283061	-2.156408	-0.320407
H	-2.215517	-0.000000	-1.527679
H	-1.283061	2.156408	-0.320407

SBV (reac'/prod'), X, 150

C	0.000432	0.727746	0.965408
C	0.809416	1.174744	-0.219276
C	1.567710	-0.000571	-0.686840
C	1.188340	-1.108912	-0.034820
C	0.005193	-0.816814	0.883692
C	-1.161548	-1.123048	-0.052680
C	-1.518865	-0.024408	-0.723208
C	-0.776165	1.155085	-0.234888
H	-0.035901	1.261269	1.906647
H	1.235612	2.170685	-0.264495
H	2.234063	0.016427	-1.544596
H	1.484831	-2.120796	-0.293476
H	-0.009185	-1.348666	1.834518
H	-1.537734	-2.123768	-0.223752
H	-2.262012	0.022116	-1.508497
H	-1.224187	2.138911	-0.286141

SBV (TS), X, 150

C	0.002564	0.776029	0.944948
C	1.021503	1.143376	-0.096761
C	1.572082	0.000000	-0.663741
C	1.021503	-1.143376	-0.096761
C	0.002564	-0.776029	0.944948
C	-0.989639	-1.136677	-0.111850
C	-1.524545	-0.000000	-0.697190
C	-0.989639	1.136677	-0.111850
H	-0.023180	1.300470	1.897174
H	1.271295	2.166855	-0.339868
H	2.191904	0.000000	-1.554867
H	1.271295	-2.166855	-0.339868
H	-0.023180	-1.300470	1.897174
H	-1.288618	-2.154497	-0.317263
H	-2.227290	-0.000000	-1.519365
H	-1.288618	2.154497	-0.317263

SBV (reac'), Y, 50

C	-0.000000	0.836420	0.876852
C	1.177500	1.113784	-0.054397
C	1.538255	-0.003050	-0.695573
C	0.790124	-1.169783	-0.190655
C	-0.000000	-0.706029	0.993455
C	-0.790124	-1.169783	-0.190655
C	-1.538255	-0.003050	-0.695573
C	-1.177500	1.113784	-0.054397
H	0.000000	1.386108	1.818451
H	1.514456	2.116399	-0.294130
H	2.245527	-0.058657	-1.514119
H	1.226096	-2.159845	-0.219790
H	-0.000000	-1.221335	1.945029
H	-1.226096	-2.159845	-0.219790
H	-2.245527	-0.058657	-1.514119
H	-1.514456	2.116399	-0.294130

SBV (TS), Y, 50

C	0.000000	0.776739	0.949525
C	0.995764	1.141436	-0.107550
C	1.540119	-0.000466	-0.683128
C	1.003546	-1.137939	-0.103542
C	0.000000	-0.774568	0.947895
C	-1.003546	-1.137939	-0.103542
C	-1.540119	-0.000466	-0.683128
C	-0.995764	1.141436	-0.107550
H	-0.000000	1.300234	1.903143
H	1.275859	2.162228	-0.326473
H	2.199586	-0.004850	-1.541371
H	1.275575	-2.159133	-0.327649
H	0.000000	-1.304958	1.896460
H	-1.275575	-2.159133	-0.327649
H	-2.199586	-0.004850	-1.541371
H	-1.275859	2.162228	-0.326473

SBV (prod'), Y, 50

C	0.000000	0.708390	0.990484
C	0.790068	1.170114	-0.194937
C	1.540760	0.000336	-0.696767
C	1.173815	-1.114171	-0.056326
C	-0.000000	-0.833769	0.877214
C	-1.173815	-1.114171	-0.056326
C	-1.540760	0.000336	-0.696767
C	-0.790068	1.170114	-0.194937
H	0.000000	1.221047	1.944222
H	1.231826	2.159546	-0.217312
H	2.244460	0.045074	-1.518859
H	1.509236	-2.118042	-0.287265
H	-0.000000	-1.387544	1.814830
H	-1.509236	-2.118042	-0.287265
H	-2.244460	0.045074	-1.518859
H	-1.231826	2.159546	-0.217312

SBV (reac'), Y, 100

C	0.000000	0.822076	0.877226
C	1.179426	1.118865	-0.046389
C	1.537243	0.015255	-0.712963
C	0.790582	-1.161139	-0.232987
C	0.000001	-0.723219	0.960427
C	-0.790581	-1.161139	-0.232987
C	-1.537243	0.015254	-0.712962
C	-1.179426	1.118865	-0.046389
H	0.000000	1.348211	1.833128
H	1.516852	2.126696	-0.267949
H	2.246264	-0.027312	-1.530944
H	1.223493	-2.151125	-0.287792
H	0.000001	-1.261696	1.898875
H	-1.223491	-2.151126	-0.287792
H	-2.246264	-0.027313	-1.530943
H	-1.516853	2.126695	-0.267948

SBV (TS), Y, 100

C	0.000000	0.773730	0.946584
C	1.007804	1.136479	-0.101781
C	1.540806	0.000927	-0.683237
C	0.992295	-1.143449	-0.109831
C	0.000000	-0.778084	0.949799
C	-0.992295	-1.143449	-0.109831
C	-1.540806	0.000927	-0.683237
C	-1.007804	1.136479	-0.101781
H	0.000000	1.307668	1.892771
H	1.276606	2.158323	-0.327574
H	2.200566	0.009718	-1.541257
H	1.277339	-2.164497	-0.325118
H	0.000000	-1.298317	1.906038
H	-1.277339	-2.164497	-0.325118
H	-2.200566	0.009718	-1.541257
H	-1.276606	2.158323	-0.327574

SBV (prod’), Y, 100

C	-0.000000	0.709689	0.990219
C	0.790409	1.170652	-0.195591
C	1.542207	-0.000532	-0.696171
C	1.172092	-1.114420	-0.056409
C	0.000000	-0.832745	0.878350
C	-1.172092	-1.114420	-0.056409
C	-1.542207	-0.000532	-0.696171
C	-0.790409	1.170652	-0.195591
H	-0.000000	1.221223	1.945096
H	1.234725	2.160473	-0.214630
H	2.243623	0.038963	-1.520433
H	1.506071	-2.119409	-0.283914
H	0.000000	-1.388917	1.814000
H	-1.506071	-2.119409	-0.283914
H	-2.243623	0.038963	-1.520433
H	-1.234725	2.160473	-0.214630

SBV (reac’), Y, 150

C	0.000000	0.841092	0.876302
C	1.181492	1.113903	-0.053061
C	1.536068	-0.007289	-0.693311
C	0.791269	-1.170209	-0.183149
C	0.000000	-0.702618	0.998420
C	-0.791269	-1.170209	-0.183149
C	-1.536068	-0.007289	-0.693311
C	-1.181492	1.113903	-0.053061
H	-0.000000	1.388623	1.821405
H	1.517310	2.116525	-0.305737
H	2.245401	-0.075105	-1.509456
H	1.220551	-2.162680	-0.218300
H	0.000000	-1.220276	1.948472
H	-1.220551	-2.162680	-0.218300
H	-2.245401	-0.075105	-1.509456
H	-1.517310	2.116525	-0.305737

SBV (TS), Y, 150

C	-0.000000	0.779613	0.949666
C	0.989158	1.145622	-0.112322
C	1.541963	-0.001379	-0.683416
C	1.012281	-1.135226	-0.100162
C	0.000000	-0.773053	0.944962
C	-1.012281	-1.135226	-0.100162
C	-1.541963	-0.001379	-0.683416
C	-0.989158	1.145622	-0.112322
H	-0.000000	1.296768	1.908555
H	1.279849	2.167238	-0.323167
H	2.202214	-0.014622	-1.541062
H	1.278325	-2.158020	-0.327117
H	0.000000	-1.310554	1.888905
H	-1.278325	-2.158020	-0.327117
H	-2.202214	-0.014622	-1.541062
H	-1.279849	2.167238	-0.323167

SBV (prod’), Y, 150

C	0.000000	0.710966	0.991440
C	0.790984	1.171679	-0.194456
C	1.543822	-0.000678	-0.694279
C	1.170459	-1.114504	-0.055762
C	0.000000	-0.832000	0.880350
C	-1.170459	-1.114504	-0.055762
C	-1.543822	-0.000678	-0.694279
C	-0.790984	1.171679	-0.194456
H	-0.000000	1.221212	1.947645
H	1.237620	2.162415	-0.209888
H	2.242653	0.033931	-1.520936
H	1.502676	-2.120844	-0.280671
H	0.000000	-1.391076	1.813889
H	-1.502676	-2.120844	-0.280671
H	-2.242653	0.033931	-1.520936
H	-1.237620	2.162415	-0.209888

SBV (reac’/prod’), Z, 50

C	-0.000000	0.816220	0.888455
C	1.175976	1.114698	-0.038316
C	1.537632	0.013211	-0.704311
C	0.789233	-1.164916	-0.224157
C	-0.000000	-0.728201	0.971834
C	-0.789233	-1.164916	-0.224157
C	-1.537632	0.013211	-0.704311
C	-1.175976	1.114698	-0.038316
H	-0.000000	1.349470	1.840058
H	1.501994	2.122931	-0.265798
H	2.231029	-0.018243	-1.535314
H	1.224226	-2.154818	-0.280358
H	-0.000000	-1.263216	1.913759
H	-1.224226	-2.154818	-0.280358
H	-2.231029	-0.018243	-1.535314
H	-1.501994	2.122931	-0.265798

SBV (TS), Z, 50

C	-0.000000	0.775776	0.952412
C	1.001250	1.139292	-0.100849
C	1.540060	0.000000	-0.679950
C	1.001250	-1.139292	-0.100849
C	0.000000	-0.775776	0.952412
C	-1.001250	-1.139292	-0.100849
C	-1.540060	0.000000	-0.679950
C	-1.001250	1.139292	-0.100849
H	-0.000000	1.302510	1.905042
H	1.267081	2.159807	-0.335757
H	2.189360	0.000000	-1.545493
H	1.267081	-2.159807	-0.335757
H	0.000000	-1.302510	1.905042
H	-1.267081	-2.159807	-0.335757
H	-2.189360	-0.000000	-1.545493
H	-1.267081	2.159807	-0.335757

SBV (reac’/prod’), Z, 100

C	-0.000000	0.729145	0.977585
C	0.788467	1.164546	-0.220455
C	1.535437	-0.013470	-0.702082
C	1.176460	-1.114394	-0.033407
C	-0.000000	-0.815547	0.893764
C	-1.176460	-1.114394	-0.033407
C	-1.535437	-0.013470	-0.702082
C	-0.788467	1.164546	-0.220455
H	-0.000000	1.265402	1.920151
H	1.219666	2.155401	-0.286566
H	2.216310	0.016316	-1.543238
H	1.491639	-2.122948	-0.274528
H	-0.000000	-1.349905	1.846643
H	-1.491639	-2.122948	-0.274528
H	-2.216310	0.016316	-1.543238
H	-1.219666	2.155401	-0.286566

SBV (TS), Z, 100

C	0.000000	0.776024	0.955946
C	1.003186	1.139082	-0.096142
C	1.540108	-0.000000	-0.677008
C	1.003186	-1.139082	-0.096142
C	-0.000000	-0.776024	0.955946
C	-1.003186	-1.139082	-0.096142
C	-1.540108	0.000000	-0.677008
C	-1.003186	1.139082	-0.096142
H	0.000000	1.302197	1.910844
H	1.258863	2.159046	-0.344343
H	2.179158	-0.000000	-1.550015
H	1.258863	-2.159046	-0.344343
H	-0.000000	-1.302197	1.910844
H	-1.258863	-2.159046	-0.344343
H	-2.179158	0.000000	-1.550015
H	-1.258863	2.159046	-0.344343

SBV (reac’/prod’), Z, 150

C	0.000000	0.730108	0.983515
C	0.787669	1.164277	-0.216801
C	1.532905	-0.013810	-0.700219
C	1.177111	-1.114150	-0.028400
C	0.000000	-0.814912	0.899160
C	-1.177111	-1.114150	-0.028400
C	-1.532905	-0.013810	-0.700219
C	-0.787669	1.164277	-0.216801
H	0.000000	1.267347	1.927272
H	1.215257	2.155937	-0.292605
H	2.201029	0.014371	-1.551683
H	1.481207	-2.122779	-0.283405
H	0.000000	-1.350237	1.853874
H	-1.481207	-2.122779	-0.283405
H	-2.201029	0.014371	-1.551683
H	-1.215257	2.155937	-0.292605

SBV (TS), Z, 150

C	-0.000000	0.775776	0.952412
C	1.001250	1.139292	-0.100849
C	1.540060	0.000000	-0.679950
C	1.001250	-1.139292	-0.100849
C	0.000000	-0.775776	0.952412
C	-1.001250	-1.139292	-0.100849
C	-1.540060	0.000000	-0.679950
C	-1.001250	1.139292	-0.100849
H	-0.000000	1.302510	1.905042
H	1.267081	2.159807	-0.335757
H	2.189360	0.000000	-1.545493
H	1.267081	-2.159807	-0.335757
H	0.000000	-1.302510	1.905042
H	-1.267081	-2.159807	-0.335757
H	-2.189360	-0.000000	-1.545493
H	-1.267081	2.159807	-0.335757

PL (reac’/prod’), no field

C	0.043005	2.164090	0.000000
C	1.161337	1.415117	0.000000
C	0.731091	0.003567	0.000000
C	-0.731091	-0.003567	0.000000
C	-1.161337	1.270839	0.000000
C	1.161337	-1.270839	0.000000
C	-0.043005	-2.164090	0.000000
C	-1.161337	-1.415117	0.000000
H	-0.005490	3.244570	0.000000
H	2.180402	1.774170	0.000000
H	-2.183005	1.629581	0.000000
H	2.183005	-1.629581	0.000000
H	0.005490	-3.244570	0.000000
H	-2.180402	-1.774170	0.000000

PL (TS), no field

C	-0.000000	0.000000	2.180781
C	0.000000	1.137525	1.349379
C	0.000000	0.704005	0.000000
C	-0.000000	-0.704005	0.000000
C	-0.000000	-1.137525	1.349379
C	0.000000	1.137525	-1.349379
C	0.000000	0.000000	-2.180781
C	-0.000000	-1.137525	-1.349379
H	-0.000000	0.000000	3.259899
H	0.000000	2.162373	1.701093
H	-0.000000	-2.162373	1.701093
H	0.000000	2.162373	-1.701093
H	0.000000	0.000000	-3.259899
H	-0.000000	-2.162373	-1.701093

PL (reac'/prod'), X, 50

C	-0.066785	2.164116	0.000001
C	1.147046	1.285545	-0.000000
C	0.734891	0.004501	-0.000003
C	-0.727270	-0.004413	-0.000003
C	-1.175641	1.400876	-0.000001
C	1.178199	-1.403664	-0.000001
C	0.066818	-2.162971	0.000001
C	-1.147583	-1.281664	-0.000000
H	-0.034920	3.245131	0.000002
H	2.164796	1.657546	0.000001
H	-2.199878	1.744279	-0.000000
H	2.200257	-1.755692	-0.000000
H	0.025548	-3.243862	0.000002
H	-2.165480	-1.649725	0.000001

PL (TS), X, 50

C	0.001341	2.180750	0.000000
C	1.137557	1.352086	-0.000000
C	0.709286	0.000014	0.000000
C	-0.699083	-0.000008	0.000000
C	-1.137608	1.346566	-0.000000
C	1.137603	-1.352116	0.000000
C	0.001301	-2.180737	-0.000000
C	-1.137555	-1.346523	0.000000
H	-0.004599	3.259879	-0.000000
H	2.161690	1.708047	0.000000
H	-2.163534	1.694157	0.000000
H	2.161742	-1.708064	-0.000000
H	-0.004679	-3.259867	0.000000
H	-2.163463	-1.694182	-0.000000

PL (reac'/prod'), X, 100

C	-0.066308	2.164873	0.000000
C	1.147111	1.287466	0.000000
C	0.738716	0.004385	-0.000001
C	-0.723467	-0.004220	-0.000001
C	-1.174389	1.399745	-0.000001
C	1.179493	-1.405332	-0.000001
C	0.066393	-2.162595	0.000000
C	-1.148211	-1.279698	0.000000
H	-0.038969	3.245950	0.000001
H	2.165391	1.661384	0.000002
H	-2.200155	1.738928	-0.000001
H	2.200932	-1.761733	-0.000001
H	0.020214	-3.243419	0.000001
H	-2.166751	-1.645733	0.000002

PL (TS), X, 100

C	0.001323	2.180805	-0.000000
C	1.137612	1.352173	0.000000
C	0.709294	-0.000001	-0.000000
C	-0.699053	0.000001	0.000000
C	-1.137599	1.346607	0.000000
C	1.137609	-1.352176	-0.000000
C	0.001317	-2.180806	0.000000
C	-1.137604	-1.346603	-0.000000
H	-0.004665	3.259936	0.000000
H	2.161747	1.708133	-0.000000
H	-2.163523	1.694208	-0.000000
H	2.161743	-1.708138	0.000000
H	-0.004674	-3.259938	-0.000000
H	-2.163529	-1.694200	0.000000

PL (reac'/prod'), X, 150

C	-0.065807	2.165739	-0.000003
C	1.147195	1.289541	-0.000002
C	0.742547	0.004258	-0.000003
C	-0.719663	-0.004034	-0.000003
C	-1.173355	1.398622	-0.000004
C	1.180980	-1.407021	0.000001
C	0.065987	-2.162358	0.000005
C	-1.148922	-1.277875	0.000003
H	-0.043046	3.246885	-0.000003
H	2.166389	1.665481	-0.000000
H	-2.200975	1.733523	-0.000006
H	2.202200	-1.767662	0.000001
H	0.014892	-3.243115	0.000009
H	-2.168423	-1.641981	0.000006

PL (TS), X, 150

C	0.003953	2.181151	-0.000000
C	1.138611	1.357802	0.000000
C	0.719955	0.000009	0.000000
C	-0.689295	0.000017	0.000000
C	-1.138615	1.341180	0.000000
C	1.138591	-1.357829	0.000000
C	0.003933	-2.181141	-0.000000
C	-1.138618	-1.341182	0.000000
H	-0.014026	3.260255	-0.000000
H	2.162352	1.722351	-0.000000
H	-2.167557	1.680789	-0.000000
H	2.162333	-1.722373	-0.000000
H	-0.014054	-3.260247	-0.000000
H	-2.167563	-1.680780	-0.000000

PL (reac'/prod'), Y, 50

C	0.067298	2.164067	-0.000002
C	1.178108	1.400470	-0.000000
C	0.731182	-0.002182	0.000000
C	-0.730695	0.007741	-0.000000
C	-1.146801	1.286762	-0.000002
C	1.145547	-1.282802	0.000002
C	-0.069473	-2.163589	0.000002
C	-1.177534	-1.402339	0.000000
H	0.034348	3.246073	-0.000002
H	2.202602	1.744828	-0.000000
H	-2.165583	1.654022	-0.000002
H	2.161617	-1.657133	0.000003
H	-0.030989	-3.243983	0.000002
H	-2.199628	-1.751931	-0.000000

PL (TS), Y, 50

C	0.000000	2.182739	0.000000
C	1.139235	1.349565	0.000000
C	0.704136	0.006180	0.000000
C	-0.704136	0.006180	0.000000
C	-1.139235	1.349565	0.000000
C	1.135753	-1.349714	0.000000
C	0.000000	-2.179605	0.000000
C	-1.135753	-1.349714	0.000000
H	0.000000	3.262690	0.000000
H	2.165904	1.696568	0.000000
H	-2.165904	1.696568	0.000000
H	2.158888	-1.706298	0.000000
H	0.000000	-3.258423	0.000000
H	-2.158888	-1.706298	0.000000

PL (reac'/prod'), Y, 100

C	0.063811	2.165622	-0.000000
C	1.176779	1.400829	0.000000
C	0.731004	0.001601	0.000001
C	-0.730053	0.009710	0.000001
C	-1.148390	1.288687	0.000000
C	1.145760	-1.280821	0.000000
C	-0.068088	-2.164582	-0.000000
C	-1.175586	-1.404653	0.000000
H	0.032625	3.249088	-0.000001
H	2.202297	1.743584	0.000000
H	-2.169066	1.651450	-0.000001
H	2.161022	-1.657781	-0.000001
H	-0.025846	-3.244778	-0.000001
H	-2.196271	-1.757957	0.000000

PL (TS), Y, 100

C	-0.000000	2.185641	-0.000000
C	1.141045	1.350460	-0.000000
C	0.704158	0.012344	0.000000
C	-0.704158	0.012344	-0.000000
C	-1.141045	1.350460	0.000000
C	1.134116	-1.350797	-0.000000
C	-0.000000	-2.179391	-0.000000
C	-1.134116	-1.350797	0.000000
H	-0.000000	3.266946	-0.000000
H	2.169555	1.692891	0.000000
H	-2.169555	1.692891	-0.000000
H	2.155591	-1.712304	0.000000
H	-0.000000	-3.258384	-0.000000
H	-2.155591	-1.712304	-0.000000

PL (reac'/prod'), Y, 150

C	-0.062061	2.168084	0.000000
C	1.148682	1.292408	0.000000
C	0.729126	0.012425	0.000000
C	-0.730466	0.005062	0.000000
C	-1.176512	1.400160	0.000000
C	1.174621	-1.406145	0.000000
C	0.068265	-2.166281	0.000000
C	-1.144429	-1.280717	0.000000
H	-0.033684	3.253537	0.000000
H	2.171061	1.651555	0.000000
H	-2.203501	1.740300	0.000000
H	2.194287	-1.762385	0.000000
H	0.023295	-3.246736	0.000000
H	-2.158683	-1.661265	0.000000

PL (TS), Y, 150

C	-0.000000	2.189425	-0.000000
C	1.142880	1.351973	-0.000000
C	0.704195	0.018468	0.000000
C	-0.704195	0.018468	-0.000000
C	-1.142880	1.351973	0.000000
C	1.132571	-1.352575	-0.000000
C	0.000000	-2.180091	0.000000
C	-1.132571	-1.352575	0.000000
H	0.000000	3.272661	0.000000
H	2.173261	1.690030	0.000000
H	-2.173261	1.690030	-0.000000
H	2.152482	-1.719023	0.000000
H	-0.000000	-3.259741	0.000000
H	-2.152482	-1.719023	-0.000000

PL (reac'/prod'), Z, 50

C	-0.066838	2.162705	-0.001207
C	1.146844	1.282612	0.009421
C	0.731115	0.004370	0.064173
C	-0.731115	-0.004366	0.064173
C	-1.176846	1.401995	0.030363
C	1.176846	-1.401993	0.030442
C	0.066838	-2.162705	-0.001086
C	-1.146844	-1.282611	0.009492
H	-0.030441	3.242174	-0.057598
H	2.163402	1.650378	-0.053073
H	-2.199747	1.749297	0.007599
H	2.199748	-1.749296	0.007697
H	0.030441	-3.242177	-0.057417
H	-2.163403	-1.650380	-0.052980

PL (TS), Z, 50

C	0.000000	2.171883	-0.029740
C	1.136582	1.342768	0.034304
C	0.704854	0.000005	0.180602
C	-0.704854	0.000005	0.180602
C	-1.136582	1.342768	0.034304
C	1.136582	-1.342766	0.034379
C	0.000000	-2.171884	-0.029620
C	-1.136582	-1.342766	0.034379
H	0.000000	3.245856	-0.136330
H	2.160826	1.688364	-0.041731
H	-2.160826	1.688364	-0.041731
H	2.160826	-1.688366	-0.041634
H	0.000000	-3.245863	-0.136150
H	-2.160826	-1.688366	-0.041634

PL (reac’/prod’), Z, 100

C	-0.067771	2.160240	-0.002710
C	1.144982	1.280176	0.018766
C	0.731238	0.004370	0.129799
C	-0.731238	-0.004363	0.129799
C	-1.177452	1.400718	0.061190
C	1.177452	-1.400715	0.061269
C	0.067771	-2.160240	-0.002589
C	-1.144982	-1.280175	0.018837
H	-0.032367	3.235251	-0.116099
H	2.157931	1.641801	-0.107126
H	-2.200277	1.746267	0.015859
H	2.200277	-1.746266	0.015957
H	0.032367	-3.235257	-0.115918
H	-2.157931	-1.641806	-0.107034

PL (TS), Z, 100

C	0.000000	2.152749	-0.051517
C	1.134683	1.328676	0.063152
C	0.706492	0.000009	0.321379
C	-0.706492	0.000009	0.321379
C	-1.134683	1.328676	0.063152
C	1.134684	-1.328673	0.063226
C	0.000000	-2.152752	-0.051396
C	-1.134684	-1.328673	0.063226
H	0.000000	3.213755	-0.250275
H	2.157256	1.661111	-0.073104
H	-2.157256	1.661111	-0.073104
H	2.157256	-1.661114	-0.073011
H	0.000000	-3.213770	-0.250095
H	-2.157256	-1.661114	-0.073011

PL (reac’/prod’), Z, 150

C	0.068851	2.155957	-0.005138
C	1.178115	1.398761	0.092026
C	0.731452	-0.004181	0.196892
C	-0.731452	0.004214	0.196893
C	-1.142050	1.275688	0.027712
C	1.142050	-1.275684	0.027938
C	-0.068850	-2.155958	-0.004759
C	-1.178115	-1.398744	0.092272
H	0.034832	3.223406	-0.175816
H	2.200755	1.741615	0.024386
H	-2.149045	1.626801	-0.161065
H	2.149046	-1.626828	-0.160779
H	-0.034833	-3.223437	-0.175253
H	-2.200756	-1.741608	0.024691

PL (TS), Z, 150

C	-0.000000	2.129246	-0.069435
C	1.132489	1.311663	0.088342
C	0.708479	0.000035	0.436186
C	-0.708479	0.000035	0.436186
C	-1.132489	1.311663	0.088342
C	1.132464	-1.311636	0.088550
C	0.000000	-2.129232	-0.069109
C	-1.132464	-1.311636	0.088550
H	0.000000	3.171696	-0.350688
H	2.152794	1.627800	-0.096874
H	-2.152794	1.627800	-0.096874
H	2.152775	-1.627848	-0.096501
H	0.000000	-3.171733	-0.350174
H	-2.152775	-1.627848	-0.096501

CBD (reac’), no field

C	0.037291	-0.545621	0.769760
C	0.037291	-0.545621	-0.769760
H	0.240203	-1.395055	1.416046
H	0.240203	-1.395055	-1.416046
C	-1.318655	0.101734	0.669919
C	-1.318655	0.101734	-0.669919
H	-1.989020	0.495846	1.422893
H	-1.989020	0.495846	-1.422893
N	1.197767	0.428722	0.619468
N	1.197767	0.428722	-0.619468

CBD (TS), no field

C	-0.616113	0.037716	0.791887
C	-0.616113	0.037716	-0.791887
H	-1.317259	-0.386510	1.498458
H	-1.317259	-0.386510	-1.498458
C	-0.051574	1.319547	0.703112
C	-0.051574	1.319547	-0.703112
H	0.325576	2.007526	1.448823
H	0.325576	2.007526	-1.448823
N	0.673846	-1.286206	0.578236
N	0.673846	-1.286206	-0.578236

CBD (prod’), no field

C	0.756620	0.731729	0.786113
C	0.756620	0.731729	-0.786113
H	0.362246	1.387624	1.550080
H	0.362246	1.387624	-1.550080
C	1.442717	-0.406104	0.783248
C	1.442717	-0.406104	-0.783248
H	1.838908	-1.062055	1.546237
H	1.838908	-1.062055	-1.546237
N	-2.148694	-0.264253	0.545504
N	-2.148694	-0.264253	-0.545504

CBD (reac’), X, -50

C	-0.192666	0.509311	-0.770499
C	-0.192666	0.509311	0.770499
H	-0.361928	1.364529	-1.418750
H	-0.361927	1.364529	1.418750
C	-1.141617	-0.652913	-0.670659
C	-1.141618	-0.652913	0.670659
H	-1.559495	-1.311074	-1.422235
H	-1.559495	-1.311074	1.422235
N	1.272520	0.115483	-0.618355
N	1.272520	0.115482	0.618355

CBD (TS), X, -50

C	-0.382773	0.518094	-0.792484
C	-0.382773	0.518094	0.792484
H	-0.375409	1.337384	-1.499140
H	-0.375409	1.337384	1.499140
C	-1.182365	-0.633213	-0.703196
C	-1.182365	-0.633213	0.703196
H	-1.547762	-1.327527	-1.449303
H	-1.547762	-1.327527	1.449303
N	1.404957	0.102669	-0.577920
N	1.404957	0.102669	0.577920

CBD (prod’), X, -50

C	-0.875305	0.583665	-0.787262
C	-0.876264	0.584731	0.784671
H	-0.556696	1.281621	-1.549194
H	-0.558588	1.283724	1.546043
C	-1.367556	-0.650416	-0.784567
C	-1.368511	-0.649355	0.783047
H	-1.615418	-1.374542	-1.548760
H	-1.617297	-1.372448	1.547918
N	2.170267	0.116738	-0.544637
N	2.169660	0.117324	0.546194

CBD (reac’), X, -100

C	-0.195147	0.514691	-0.771267
C	-0.195146	0.514690	0.771269
H	-0.351109	1.370702	-1.421703
H	-0.351105	1.370699	1.421708
C	-1.147258	-0.642221	-0.671523
C	-1.147257	-0.642222	0.671525
H	-1.546068	-1.315037	-1.421317
H	-1.546067	-1.315038	1.421318
N	1.269087	0.107533	-0.617161
N	1.269089	0.107532	0.617161

CBD (TS), X, -100

C	-0.384688	0.521964	-0.792883
C	-0.384688	0.521964	0.792883
H	-0.364916	1.341357	-1.499159
H	-0.364916	1.341357	1.499159
C	-1.189492	-0.627673	-0.703310
C	-1.189492	-0.627673	0.703310
H	-1.531112	-1.334910	-1.449140
H	-1.531112	-1.334910	1.449140
N	1.386856	0.096668	-0.577770
N	1.386856	0.096668	0.577770

CBD (prod’), X, -100

C	-0.846822	0.555390	-0.784375
C	-0.845664	0.554198	0.787314
H	-0.458227	1.221069	-1.542612
H	-0.455960	1.218728	1.545987
C	-1.411744	-0.647320	-0.783444
C	-1.410588	-0.648509	0.785391
H	-1.668556	-1.366592	-1.549680
H	-1.666288	-1.368933	1.550914
N	2.215248	0.189168	-0.546414
N	2.215957	0.188462	0.544288

CBD (reac’), X, -150

C	-0.197234	0.519555	-0.772086
C	-0.197235	0.519555	0.772086
H	-0.336815	1.376607	-1.424757
H	-0.336814	1.376607	1.424757
C	-1.155005	-0.629490	-0.672629
C	-1.155005	-0.629489	0.672630
H	-1.535269	-1.316639	-1.420064
H	-1.535270	-1.316639	1.420065
N	1.266247	0.095569	-0.615762
N	1.266247	0.095568	0.615762

CBD (TS), X, -150

C	-0.385725	0.525507	-0.793060
C	-0.385725	0.525507	0.793060
H	-0.354872	1.345285	-1.498573
H	-0.354872	1.345285	1.498573
C	-1.197329	-0.621982	-0.703451
C	-1.197329	-0.621982	0.703451
H	-1.514544	-1.342324	-1.448274
H	-1.514544	-1.342324	1.448274
N	1.369118	0.090920	-0.577790
N	1.369118	0.090920	0.577790

CBD (prod’), X, -150

C	-0.784367	0.493411	-0.786862
C	-0.785685	0.494520	0.784034
H	-0.302613	1.100787	-1.540493
H	-0.305180	1.102955	1.537609
C	-1.476541	-0.641284	-0.786654
C	-1.477863	-0.640175	0.784268
H	-1.776961	-1.342731	-1.554257
H	-1.779560	-1.340547	1.552357
N	2.310470	0.319632	-0.543989
N	2.309563	0.320108	0.546616

CBD (reac’), X, 50

C	-0.190303	0.504680	-0.768766
C	-0.190639	0.504639	0.769259
H	-0.379413	1.359167	-1.412742
H	-0.380030	1.359090	1.413200
C	-1.139310	-0.662164	-0.669445
C	-1.139604	-0.662200	0.669461
H	-1.596560	-1.289630	-1.423218
H	-1.597191	-1.289699	1.423001
N	1.277520	0.129716	-0.619793
N	1.277250	0.129682	0.620905

CBD (TS), X, 50

C	-0.376603	0.509314	-0.791111
C	-0.376603	0.509314	0.791111
H	-0.397507	1.328985	-1.497403
H	-0.397507	1.328985	1.497403
C	-1.170337	-0.643918	-0.703053
C	-1.170337	-0.643918	0.703053
H	-1.581666	-1.312842	-1.447803
H	-1.581666	-1.312842	1.447803
N	1.442760	0.115869	-0.578693
N	1.442760	0.115869	0.578693

CBD (prod’), X, 50

C	-0.884100	0.617194	-0.786019
C	-0.884100	0.617194	0.786020
H	-0.693642	1.357329	-1.551213
H	-0.693642	1.357328	1.551214
C	-1.283114	-0.650554	-0.782774
C	-1.283113	-0.650554	0.782774
H	-1.549115	-1.371064	-1.543889
H	-1.549115	-1.371065	1.543888
N	2.123640	0.009133	-0.545706
N	2.123639	0.009132	0.545706

CBD (reac'), X, 100

C	-0.190726	0.500928	-0.768485
C	-0.190729	0.501128	0.768036
H	-0.388022	1.355247	-1.410428
H	-0.388029	1.355614	1.409755
C	-1.141867	-0.666269	-0.669127
C	-1.141870	-0.666094	0.668979
H	-1.619436	-1.277866	-1.423077
H	-1.619439	-1.277496	1.423087
N	1.279131	0.133284	-0.621396
N	1.279129	0.133446	0.621050

CBD (TS), X, 100

C	-0.372393	0.504315	-0.790161
C	-0.372393	0.504315	0.790161
H	-0.408985	1.324399	-1.495662
H	-0.408985	1.324399	1.495662
C	-1.165466	-0.649178	-0.703011
C	-1.165466	-0.649178	0.703011
H	-1.599145	-1.305477	-1.446295
H	-1.599145	-1.305477	1.446295
N	1.462636	0.123347	-0.579317
N	1.462636	0.123347	0.579317

CBD (prod'), X, 100

C	-0.804816	0.628800	-0.786151
C	-0.804816	0.628800	0.786151
H	-0.641106	1.374988	-1.551836
H	-0.641106	1.374988	1.551836
C	-1.227940	-0.631783	-0.781956
C	-1.227940	-0.631783	0.781956
H	-1.541483	-1.335941	-1.540247
H	-1.541483	-1.335941	1.540247
N	2.155204	-0.071618	-0.546001
N	2.155204	-0.071618	0.546001

CBD (reac'), X, 150

C	-0.195415	0.494254	-0.767470
C	-0.195524	0.494271	0.767519
H	-0.413242	1.345470	-1.407135
H	-0.413436	1.345502	1.407134
C	-1.131388	-0.687229	-0.668787
C	-1.131486	-0.687211	0.668733
H	-1.619758	-1.290034	-1.422859
H	-1.619967	-1.289993	1.422751
N	1.282245	0.155432	-0.621924
N	1.282157	0.155445	0.622177

CBD (TS), X, 150

C	-0.367379	0.498793	-0.789034
C	-0.367379	0.498793	0.789034
H	-0.420669	1.319430	-1.493264
H	-0.420669	1.319430	1.493264
C	-1.161348	-0.654495	-0.702973
C	-1.161348	-0.654495	0.702973
H	-1.617217	-1.298001	-1.444368
H	-1.617217	-1.298001	1.444368
N	1.483261	0.131679	-0.580112
N	1.483261	0.131679	0.580112

CBD (prod'), X, 150

C	-0.195415	0.494254	-0.767470
C	-0.195524	0.494271	0.767519
H	-0.413242	1.345470	-1.407135
H	-0.413436	1.345502	1.407134
C	-1.131388	-0.687229	-0.668787
C	-1.131486	-0.687211	0.668733
H	-1.619758	-1.290034	-1.422859
H	-1.619967	-1.289993	1.422751
N	1.282245	0.155432	-0.621924
N	1.282157	0.155445	0.622177
